# High-turnover copper-catalyzed amination of aryl bromides: exploring catalyst and ligand degradation pathways

**DOI:** 10.1039/d6ra05376a

**Published:** 2026-07-03

**Authors:** Tania Di Felice, Troy Yu-Ting Chen, David Sale, D. Christopher Braddock, Robert P. Davies

**Affiliations:** a Department of Chemistry, Molecular Sciences Research Hub, Imperial College London White City Campus 82 Wood Lane London W12 0BZ UK r.davies@imperial.ac.uk; b Process Studies Group, Jealott's Hill Research Centre Syngenta Bracknell Berkshire RG42 6EY UK

## Abstract

Copper-catalyzed Ullmann-type amination has emerged as a cost-effective and sustainable alternative to palladium-based C–N coupling, yet its broader adoption is often limited by high catalyst loadings. These high loadings arise in part from catalyst deactivation pathways that are still not fully understood. In this study, we examine the mechanism and stability of a homogeneous copper–oxalamide catalytic system for the coupling of aryl bromides with primary amines. As well as revealing mechanistic insight into the catalytic process, these kinetic studies show that under these conditions (EtOH solvent and KOH base) the copper centre is remarkably robust, but the oxalamide ligand undergoes rapid base-mediated hydrolysis, thus establishing ligand decomposition as a key limitation to catalyst longevity. By compensating for this ligand instability through controlled excess, we are able to achieve exceptionally low copper loadings of 5–50 ppm, delivering turnover numbers in copper of up to 7 × 10^4^ for aryl bromides and 2 × 10^5^ for aryl iodides. These findings further highlight copper's potential as a greener alternative to palladium in pharmaceutical and agrochemical synthesis and provide a foundation for further ligand design taking into account both catalyst stability and activity.

## Abstract 2

Guided by new mechanistic and kinetic insights, an improved copper‑catalysed amination protocol for aryl bromides is presented that operates at ultra‑low ppm copper loadings and delivers record turnover numbers.

## Graphical abstract

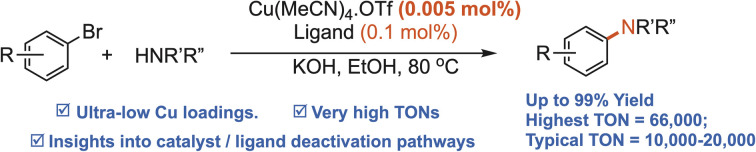

## Introduction

Aryl amines are important building blocks for a wide range of pharmaceuticals, agrochemicals, natural products, and advanced materials.^1^ Among the numerous strategies for carbon–nitrogen bond formation the Pd-catalyzed Buchwald–Hartwig reaction stands out for its efficiency and versatility, making it one of the most widely adopted methods.^2–4^ However, copper-catalyzed amination (Ullmann coupling) is of growing importance due to the low cost and low toxicity of copper and its commonly employed N, O based ligands. Additionally, copper-based systems tend to exhibit good functional group tolerance and broad applicability.^5–7^

**Scheme 1:**
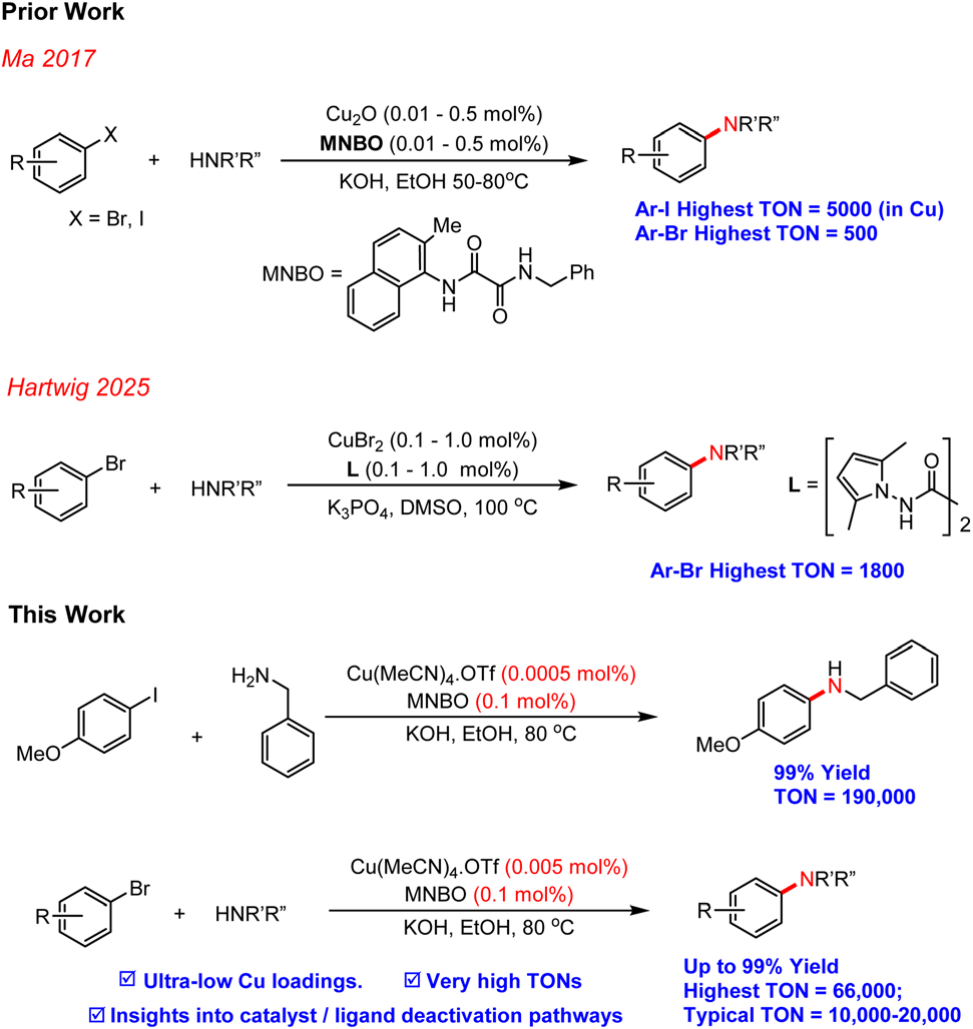
Recent examples of Cu-catalysed coupling of amines with high TONs^11,13^ and this work.

Over the last two decades, a large number of oxygen- and nitrogen-based ligands have been identified as effective promoters of copper-catalyzed C–N bond formation. Among these, oxalamide ligands, as pioneered by Ma,^8^ are perhaps the most studied and have demonstrated significant improvements in catalyst turnover number and frequency, as well as enhanced reactivity toward challenging substrates such as aryl chlorides.^7^ Although the majority of copper-based catalytic systems still require high catalyst loadings (5–10 mol%), some highly active sub-mol% systems have been reported: Norrby^9^ and Buchwald^10^ first independently demonstrated aryl iodide couplings with *N*-heterocyclic nucleophiles or phenol/thiophenol using Cu_2_O loadings as low as 0.001 mol% (10 ppm). These reactions progressed in the presence of a large excess of DMEDA (20 mol%) to give turnover numbers (TONs) of up to 86 000. For primary amine nucleophiles, Ma and co-workers have shown TONs approaching 5000 (in Cu) for couplings of (hetero)aryl iodides and bromides using 0.01 mol% Cu_2_O and a *N*-(naphthalene-1-yl)-*N*′-alkyl oxalamide ligand.^11^ More recently, Hartwig and Ray have reported on a new class of oxalohydrazide ligand for C–O bond formation from aryl bromides with TONs up to 8000 using 0.0125 mol% CuBr pre-catalyst,^12^ as well as for C–N bond formation using primary amines with 0.1 mol% of a Cu(ii) catalyst and TONs of up to 1800 (see Scheme 1).^13^

Studies on the mechanism of copper-catalyzed amination, including catalyst deactivation, have also advanced significantly. Work by our group^14–20^ and others^21–23^ has contributed to a deeper understanding of these systems, with an oxidative addition rate-limiting step at a Cu(i) centre leading to a Cu(iii) intermediate initially emerging as the favoured mechanism for most reported cases. These studies have also shown that high levels of catalyst deactivation often occur in the reaction mixture, and this is thought to be one of the main driving forces behind the requirement of high catalyst loadings.^15,17^

However, most of these kinetic and mechanistic studies to date have focused on first-generation ligands, such as phenanthroline (phen),^15^ with studies on systems employing the more active (but substantially less soluble) second-generation oxalamide ligands remaining rare. A notable exception is Hartwig's recent study on copper catalysed C–O bond formation utilising a bis-(2-phenylphenyl)oxalamide ligand. This work pointed strongly to an alternative mechanistic pathway in which the ligand is non-innocent and oxidative addition of the aryl halide occurs at a Cu(ii) centre.^24^ The key role of a Cu(ii) complex also has implications towards improved catalyst stability, a factor harnessed by the researchers to help improve TONs to up to 1000. This has more recently also been applied to C–N bond forming catalysis by the same researchers with an oxalohydrazido ligand and Cu loadings of 0.1–0.2 mol%.^13^

This study aims to deepen the current understanding of the mechanism and catalyst deactivation pathways in copper–oxalamide-catalyzed C–N bond formation between aryl bromides and primary amines. Insights gained from this investigation have been used to modify existing systems leading to enhanced catalyst performance and significantly boosted turnover numbers. In order to achieve this a fully homogeneous reaction system has been developed for kinetic studies, and Time-Adjustment Analysis^25^ and Variable Time Normalisation Analysis (VTNA)^26^ have been used to obtain mechanistic information. These studies also reveal a copper independent deactivation pathway for the ligand itself. We show how copper loadings can be lowered to as little as 5–50 ppm Cu, with significantly improved TONs achievable of up to 7 × 10^4^ with aryl-bromide substrates and 2 × 10^5^ with aryl iodides (Scheme 1).

## Results and discussion

### Kinetics: preliminary work

To obtain reliable kinetic data, efforts were initially directed toward establishing a fully homogeneous reaction system for study. Oxalamide-based ligands typically display very poor solubility in standard solvents such as dimethyl sulfoxide (DMSO), even when using elevated temperatures. However, Ma *et al.* have reported on a close to homogeneous base/solvent system comprising potassium hydroxide (KOH) and ethanol (EtOH) for the coupling of primary amines with aryl bromide substrates using oxalamide ligands.^11^ This protocol proceeds effectively with minimal catalytic loading, utilizing only 0.1 mol% of cuprous oxide (Cu_2_O) in combination with an oxalamide ligand such as *N*-(2-methylnaphthalen-1-yl)-*N*′-benzyl oxalamide (MNBO).

**Scheme 2:**
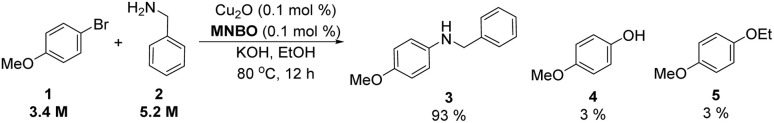
Observed products and corresponding yields from the model copper-catalyzed bond formation reaction.

In our hands we were able to reproduce Ma's results, doubling the amount of EtOH solvent used to ensure full dissolution of the base. Analysis of the product mixture by GC-MS and ^1^H NMR (see SI) revealed a yield of the coupled product 3 of 93% as well as the formation of two minor by-products: 4-methoxyphenol (4, ∼3% yield) and 1-ethoxy-4-methoxybenzene (5, ∼3% yield) (Scheme 2). These by-products have been reported in the literature in related coupling reactions and can be attributed to copper-catalysed side reactions of the aryl bromide with water or *in situ*–generated potassium ethoxide respectively.^18,27^ Given their low yield, these species were considered to have negligible impact on the reaction kinetics.

Additionally, a small quantity of white solid was observed to precipitate over the course of the reaction. This was isolated and identified *via* powder X-ray diffraction (PXRD) as potassium bromide (KBr) (see SI). Although this means the reaction mixture was not entirely homogeneous, the KBr precipitate was deemed kinetically not relevant.

The limited solubility of the copper precursor Cu_2_O also made reproducibility challenging, particularly during the preparation of stock solutions. Consequently, alternative copper sources were investigated. Copper(i) iodide showed similarly poor solubility in ethanol, but dissolved completely in acetonitrile. Under these conditions, it delivered comparable yields despite providing only half the molar amount of copper relative to Cu_2_O. However, concentration-time profiles showed an approximate induction period of up to 1 hour when copper(i) iodide was used as a stock solution in acetonitrile (see SI). One plausible explanation for this observation is that the excess of acetonitrile slows coordination of the oxalamide ligand, thereby delaying formation of the active catalytic species (note that no induction period was observed when using solid Cu^I^I). By contrast, Cu^I^(MeCN)_4_OTf demonstrated excellent solubility in ethanol and did not exhibit any detectable induction period, yielding consistent and reproducible concentration profiles (see SI), thus making it a practical alternative for kinetic studies.

An additional challenge encountered during method development was the progressive change in reflux temperature over the course of the reaction. Due to the higher boiling points of the starting materials compared to ethanol (1, 223 °C; 2 185 °C; ethanol 78.5 °C), the initial reflux temperature of the reaction mixture was measured at 112 °C, and this subsequently decreased to 90 °C where it plateaued as the reaction reached completion (see SI). Hence, in order to maintain a stable temperature diethylene glycol monoethyl ether (DEGEE, boiling point 193 °C), was used as the solvent and the reaction temperature set to a consistent 105 °C. This gave similar yields to the ethanol system (3 96%; 4, 2%; 5, 2%) and was thus employed in the kinetic studies for assessing the rate in substrates. For catalyst deactivation studies, ethanol was used as the solvent to maintain alignment with Ma's original experimental conditions.^11^

### Reaction order in substrates and catalyst

**Fig. 1:**
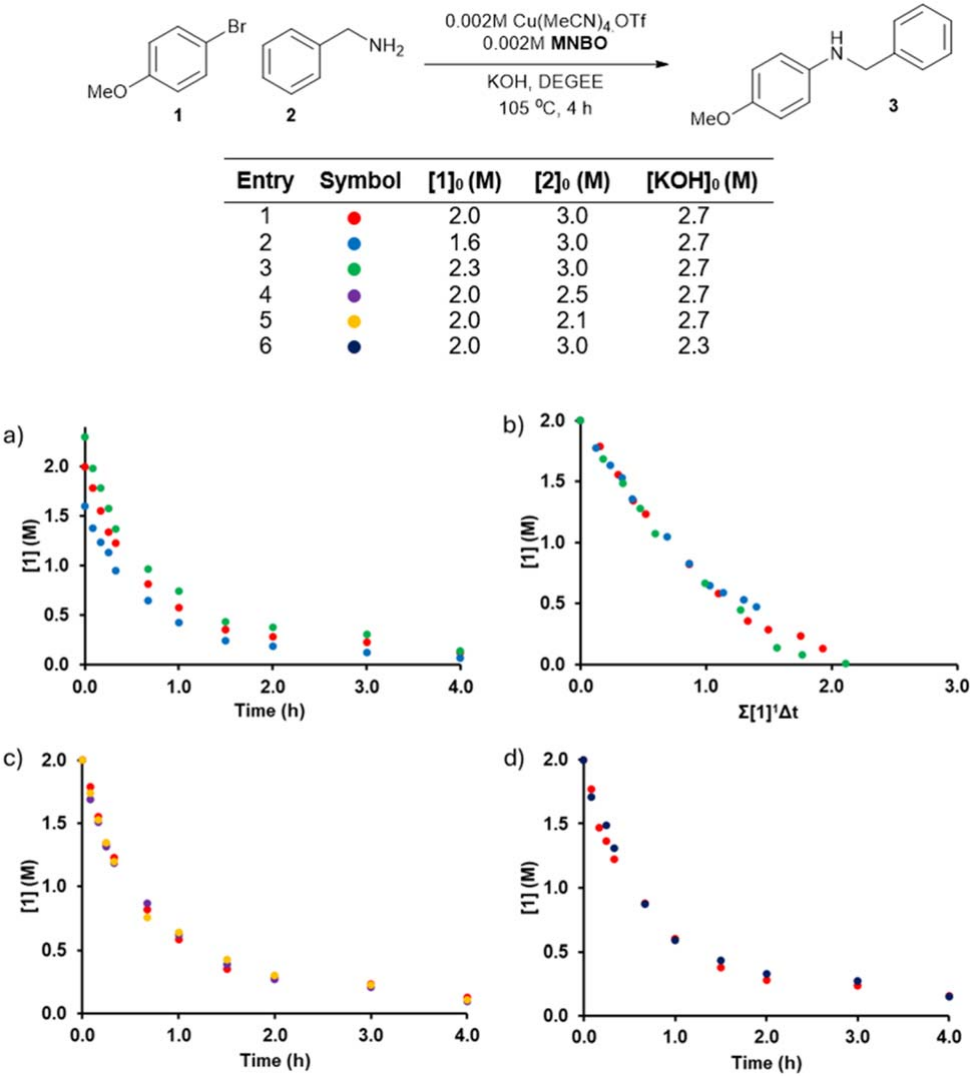
(a) Time course analysis for different excess in aryl bromide 1 (table entries 1–3); (b) VTNA analysis showing first order in [1] (table entries 1–3); (c) time course plot for different excess in amine experiment showing zero order in [2] (table entries 1, 4 and 5); (d) time course plot for different excess in base experiment showing zero order in [KOH] (table entries 1 and 6).

The ‘different excess’ protocol^25,26^ was employed to determine the rate dependence in each of the substrates: amine, aryl bromide, and KOH. Firstly, the reaction profile was obtained using three different starting concentrations of aryl halide 1, all other reaction conditions being kept constant (Fig. 1a). When the reaction profiles were normalized by assuming a first-order dependence on aryl halide 1, all three curves overlaid closely (Fig. 1b). This indicates that the reaction is first order in aryl halide in accordance with VTNA methodology.^26^ This finding is in alignment with the majority of prior kinetic studies on the Ullmann C–N cross-coupling reaction, and is consistent with aryl halide activation being the rate-determining step.^15,17,18,23^

Varying the starting concentrations of amine 2 and KOH individually yielded reaction profiles that directly overlaid with the standard reaction profile without any normalisation (Fig. 1c and d). This behaviour is indicative of zero order kinetics with respect to both components. Similar zero order dependence on NaOH and the amine nucleophile has been observed in our studies involving tartramide ligands.^18^ In addition investigations by Blackmond^23^ and Hartwig^24^ on related systems featuring anilines and potassium phenoxide respectively as nucleophiles, also observed zero order kinetics in nucleophile. In each of case, the kinetic behaviour was attributed to an aryl iodide first pathway wherein oxidative addition of the aryl halide precedes attack by the nucleophile.

**Fig. 2:**
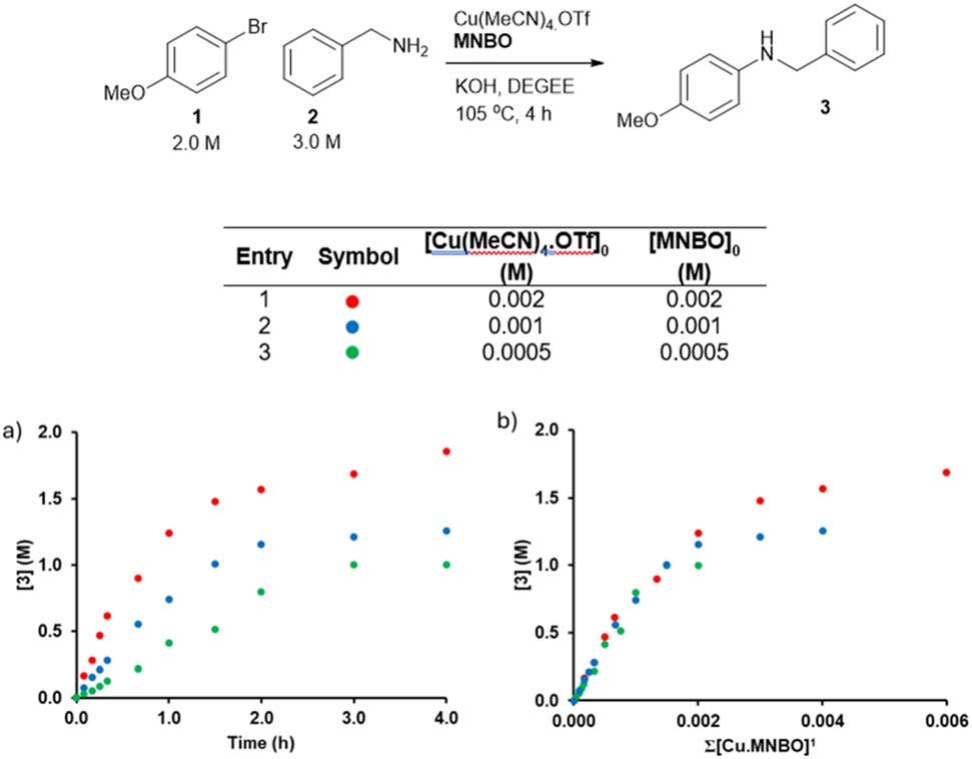
(a) Time course analysis for different excess in pre-catalyst; (b) VTNA analysis showing first order in [Cu·MNBO] in the initial phase of the reaction.

The reaction order with respect to the catalyst was then determined, assuming a 1 : 1 Cu : ligand ratio in the active catalytic species. When the reaction profiles were normalised to assume a first order dependence on catalyst concentration, a good overlap was observed in the initial phase of the reaction (Fig. 2). This indicates a first order dependence on catalyst, and is again in alignment with previous studies.^15–18,23,24^ However as the reaction progressed the curves began to diverge and no longer overlap. This deviation may be attributed to catalyst decomposition, a possibility that is examined in more detail below.

### Catalyst deactivation

**Fig. 3:**
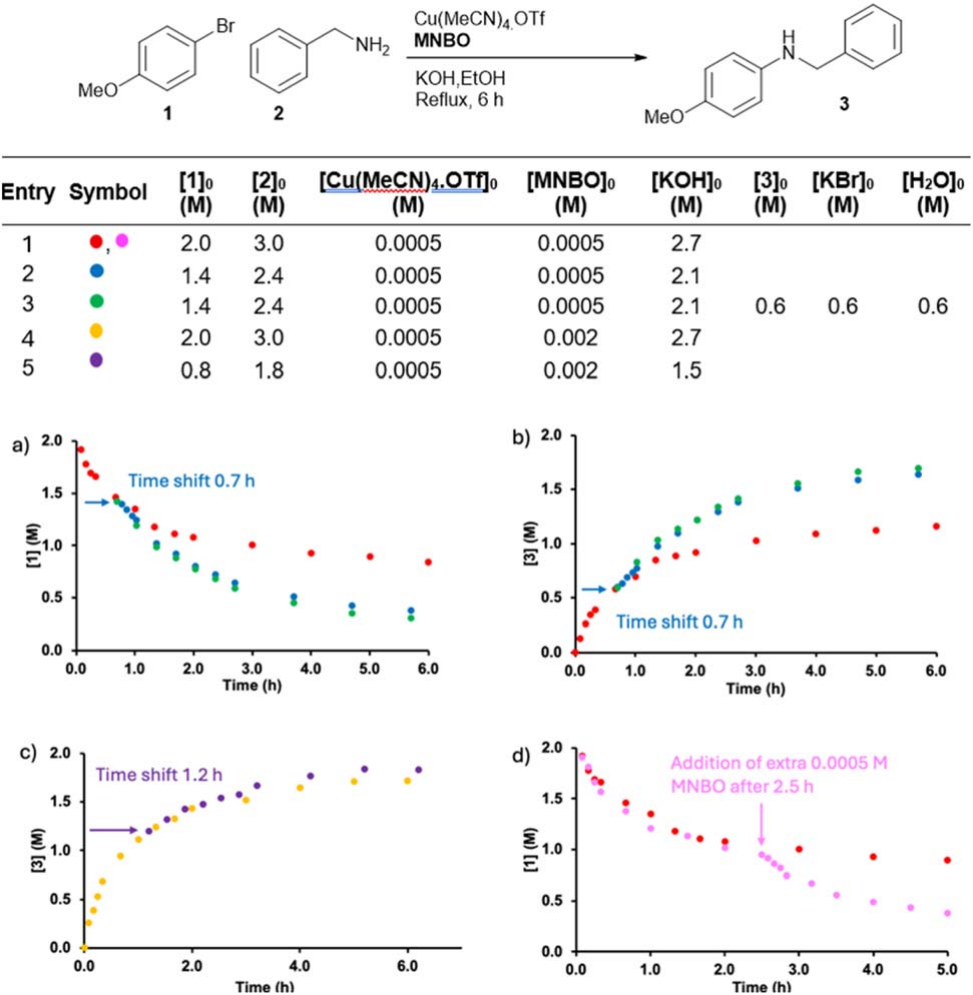
Time-shifted reaction course analysis for same excess in pre-catalyst for (a) consumption of 1 and (b) formation of 3 showing catalyst deactivation and no-product inhibition. (c) Time-shifted reaction course analysis for same excess in pre-catalyst with increased [MNBO] showing a significant reduction in catalyst deactivation. (d) Reaction course analysis showing reaction re-initiation after addition of second aliquot of MNBO.

In order to investigate potential catalyst deactivation pathways, same-excess experiments were conducted in accordance with the Blackmond RPKA protocol.^25^ These experiments were performed at reduced [Cu] and [MNBO] loadings (0.005 M, 0.025 mol%), where higher turnover numbers were expected to accentuate any deactivation effects. Indeed, significant catalyst deactivation was observed, as evidenced by the divergence in time-shifted reaction profiles for both the consumption of aryl bromide 1 and the formation of cross-coupled product 3 (Fig. 3a and b). Moreover, the addition of the reaction product (3) and side products (KBr and H_2_O) did not alter the reaction trajectory, indicating that the observed lower reaction rate is not due to product inhibition.

Increasing the concentration of the oxalamide ligand ([MNBO] = 4[Cu]) substantially mitigated catalyst deactivation, as evidenced by the now overlapping traces in a new same excess experiment (Fig. 3c). This indicates that a larger ligand reservoir is necessary for sustained catalytic turnover. Further support for this hypothesis came from the re-initiation of the reaction upon addition of a second aliquot of the ligand after the reaction profile had plateaued (Fig. 3d).

**Fig. 4:**
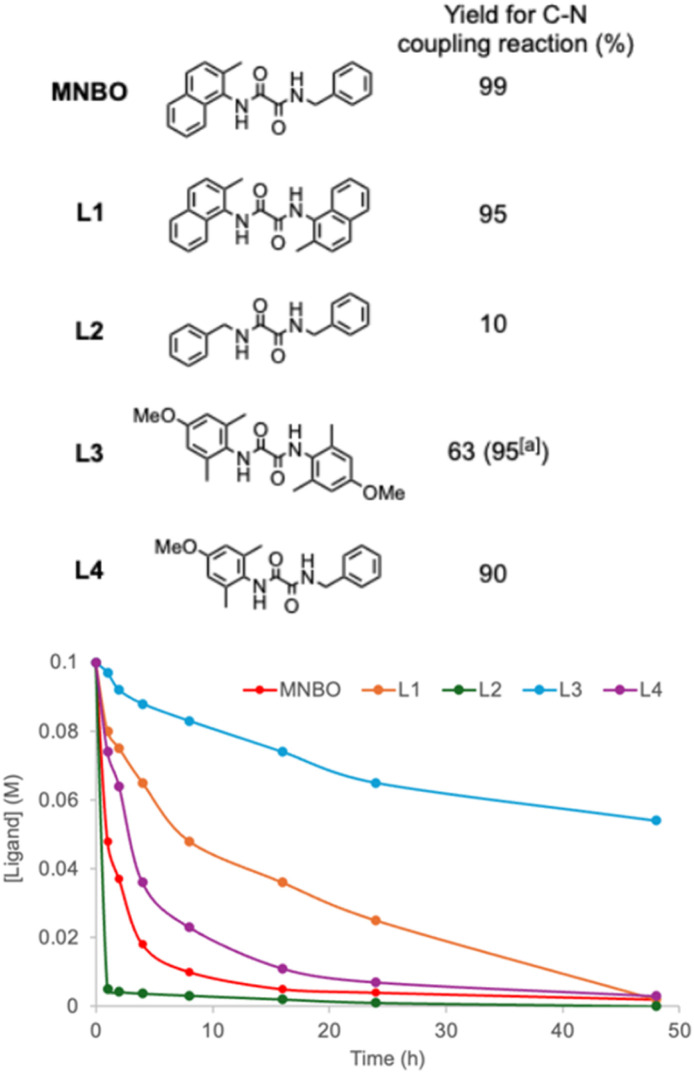
Degradation profiles for oxalamide ligands (0.1 mmol ligand, 1.3 mmol KOH, 1 mL EtOH, 80 °C) as determined using ^1^H NMR with 1,3,5-trimethoxybenzene as an internal standard. Catalytic C–N coupling yields are for the reaction of 4-bromoanisole (1, 10 mmol) with benzylamine (2, 15 mmol), using Cu(i)(MeCN)_4_OTf (0.005 mol%), ligand (0.1 mol%), KOH (13 mmol), and ethanol (1.0 mL), at 80 °C for 48 h (except ^[*a*]^ 144 hours]. Yields determined using ^1^H NMR with naphthalene as an internal standard.

These experiments indicate that the ligand is either consumed or transformed into an inactive species during the reaction. This transformation, rather than copper deactivation, appears to be the main factor contributing to catalyst deactivation. To investigate this further, a sample of the MNBO ligand (0.1 mmol) was treated with KOH (1.3 mmol) in ethanol at 80 °C under conditions analogous to those employed in the catalytic reaction (Scheme 2). ^1^H NMR analysis of the resulting solution revealed near-complete hydrolysis of the ligand in 24 hours (Fig. 4).

Comparative degradation studies on related oxalamide ligands revealed substantial variation in hydrolytic stability, with *ortho*-substituted aryl substituents offering significantly greater resistance to base-mediated hydrolysis than benzyl group substituents (Fig. 4). For MNBO, 2-methyl-1-naphthylamine was the only identifiable decomposition product, with benzylamine and oxalic acid likely by-products which are lost during aqueous workup. In the case of the symmetric ligand L1, the amount of 2-methyl-1-naphthylamine produced during hydrolysis is double the ligand consumption thus confirming full hydrolysis of both amide functionalities (see SI). The presence of copper had minimal influence on ligand decomposition rates (see SI), whereas omission of the base suppressed hydrolysis almost entirely. Although oxalamide hydrolysis has been observed previously in copper-catalysed systems using DMSO solvent and K_3_PO_4_ base, the extent of decomposition was substantially lower and did not noticeably impact the reaction kinetics, in contrast to the behaviour observed here.^28^

No direct correlation between hydrolysis rate of the ligand and its catalytic performance could be drawn (Fig. 4). Nevertheless, ligands that underwent hydrolysis most rapidly (such as L2) generally produced very low conversions. Interestingly, the ligand exhibiting the greatest hydrolytic stability (L3) was not the most catalytically efficient, reaching only 63% conversion after 48 hours. However, extending the reaction duration to seven days increased the yield to 95%, consistent with the decomposition studies which showed a substantial amount of ligand (>50%) still remained in solution after the initial 48-hour period (Fig. 4). These results demonstrate that achieving a balance between hydrolytic stability and ligand activity is key to obtaining the highest catalytic performance.

### Reaction optimisation and scope

To maximize turnover numbers under ppm-level copper loadings, we systematically optimized reaction conditions for the model Ullmann-type C–N coupling with MNBO ligand as this was shown to be the most effective ligand under the conditions studied (Fig. 4). The baseline conditions (0.005 mol% (50 ppm) Cu, 0.1 mol% ligand; Table 1, entry 1), afforded TONs approaching 2 × 10^4^, significantly exceeding previously reported TONs by an order of magnitude (Scheme 1). Control experiments confirmed that omission of copper or substitution with palladium resulted in no product formation (entries 2 and 3), excluding the possibility of trace contaminants as the active species.

**Table 1:** Evaluation of reaction parameters for copper-catalysed C–N coupling reaction

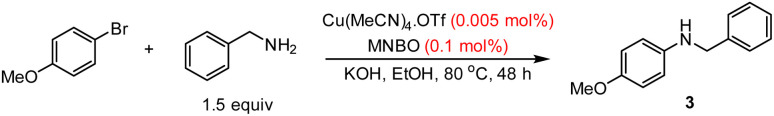			
Entry	Deviation from standard conditions	Yield (%)	TON
1	None	99	19 800
2	No Cu	0	0
3	Pd(OAc)_2_ instead of Cu	0	0
4	24 h instead of 48 h	89	17 750
5	12 h instead of 48 h	83	16 660
6	0.0025 mol% Cu	52	2600
7	0.0025 mol% Cu and 144 h	92	36 600
8	0.0005 mol% Cu	7	13 250
9	0.0005 mol% Cu, 144 h	32	66 000
10	0.05 mol% ligand	9	1750
11	0.025 mol% ligand	4	800
12	No ligand	0	0
13	K_3_PO_4_ instead of KOH	0	0

Reducing the reaction time led to lower yields and TONs (entries 4 and 5), although the 12 h experiment (entry 5) exhibited the highest observed turnover frequency (TOF ≈ 1400 h^−1^) with a respectable yield of 83%. Lowering copper loading to 0.0025 mol% decreased conversion (entry 6), but extending the reaction to 144 h restored high yield (92%), signifying the long lifetime of the catalytic system. At 0.0005 mol% (5 ppm) Cu loading initial yields were poor (7%, entry 8), yet prolonged reaction times improved conversion to 36% (entry 9), which corresponded to the highest TON recorded here (66 000). Reducing ligand concentration caused a sharp decline in yield (entries 10 and 11), underscoring the necessity of excess ligand in the system. Omission of the ligand (entry 12) or replacement of KOH by a milder base such as K_3_PO_4_ (entry 13) gave no product.

**Fig. 5:**
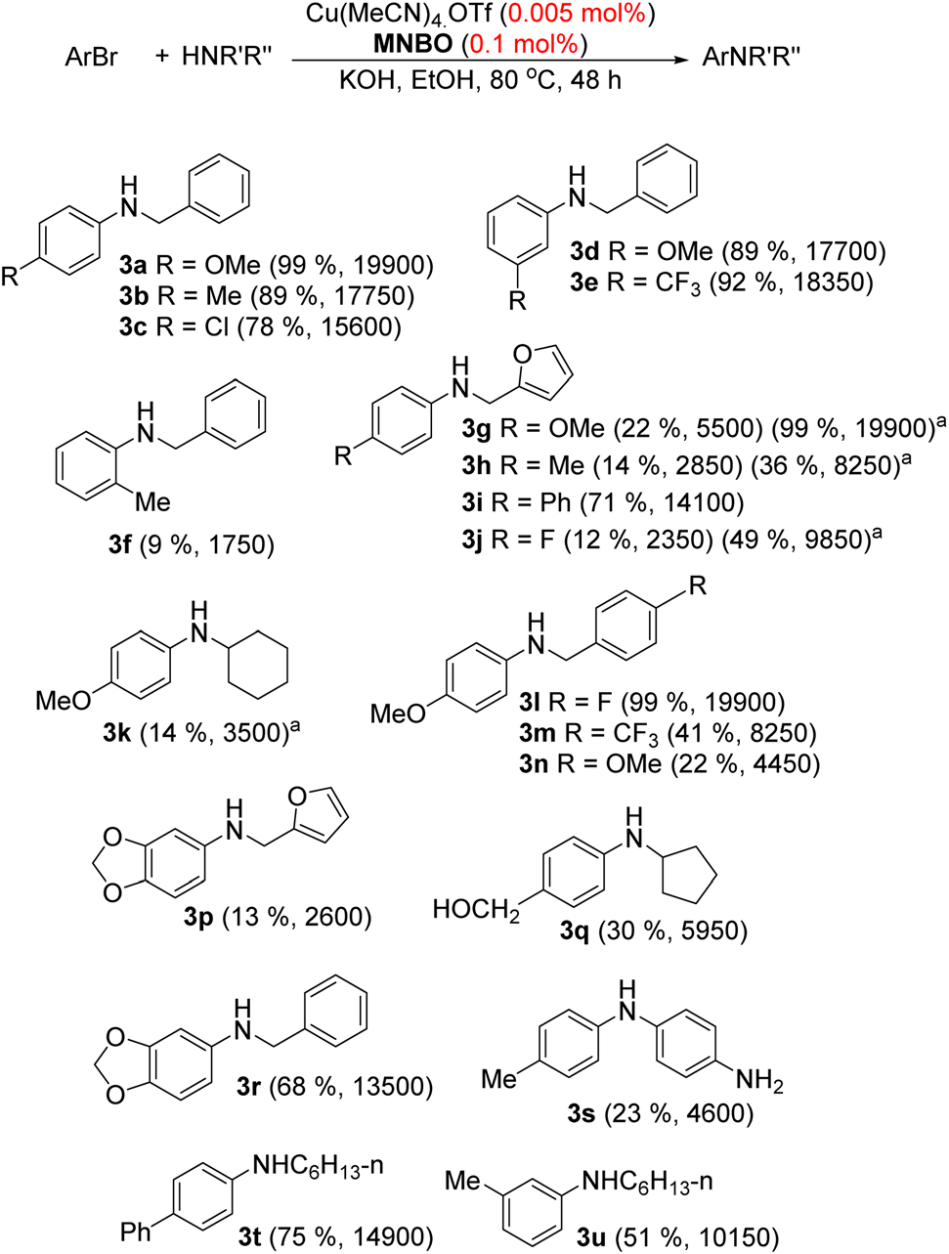
Substrate scope for Ullmann C–N coupling with 0.005 mol% (50 ppm) Cu. Yields are reported as NMR yields using naphthalene as an internal standard. Reactions performed at a scale of 5 mmol based on aryl bromide substrate. ^*a*^96 h and 1 mol% MNBO loading.

A broad substrate scope was examined under the optimized conditions (0.005 mol% = 50 ppm Cu, 0.1 mol% ligand), delivering good yields and high turnover numbers (typically exceeding 1 × 10^4^) within practical reaction times (Fig. 5). Both electron-rich and electron-deficient aryl bromides bearing *para*- and *meta*-substituents coupled efficiently with primary amines, affording products in >70% yield (entries 3a–3e, 3r). In contrast, *ortho*-substituted aryl bromides exhibited negligible reactivity, even upon increasing ligand loading and extending reaction time, likely due to steric hindrance (entry 3f). Reactions employing furfurylamine were less effective than those with benzylamine (entries 3g–3j, 3p), although yields improved with a longer reaction time and higher ligand concentrations. Functionalized benzylamines bearing electron-withdrawing substituents provided high or moderate yields (entries 3l, 3m), whereas electron-donating groups such as –OMe resulted in diminished yields (entry 3n). Amines with (hetero)cyclic substituents, including five- and six-membered rings, displayed poor reactivity (entries 3k, 3q), as did less nucleophilic substrates such as *p*-phenylenediamine (entry 3s). In contrast, aliphatic amines such as hexylamine coupled efficiently with both *para*- and *meta*-substituted aryl bromides (entries 3t, 3u). All substrate scope reactions reported here were optimised to achieve high TON with ultra-low Cu loadings. For lower-yielding substrates, previous reports have shown how higher yields can be obtained by increasing copper loadings albeit with a substantial reduction in TON.^11^

**Fig. 6:**
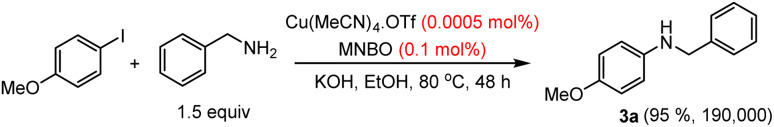
Ullmann C–N coupling for aryl iodide with 0.0005 mol% (5 ppm) Cu. NMR yield using naphthalene as an internal standard.

Replacement of the aryl bromide with a more reactive aryl iodide led to superior reactivity, delivering near-quantitative conversion (95% yield) at only 0.0005 mol% (5 ppm) Cu loading (0.1 mol% MNBO) and achieving, to the best of our knowledge, the highest TON for copper catalysed amination reported to date (1.9 × 10^5^) and a TOF of 4000 h^−1^ (Fig. 6). This was again contingent on the use of a large excess of ligand as repeating the experiment with reduced amount of MNBO (0.01 mol%) gave a lower yield of 55% and a TON of 1.1 × 10^5^.

A larger-scale preparative reaction (10 mmol of the aryl bromide) conducted under identical catalyst and ligand loadings (0.005 mol% Cu and 0.1 mol% MNBO) afforded compound 3a in 88% isolated yield.

### Mechanism

The mechanistic and kinetic data obtained in this work mainly aligns with prior studies using first generation ligands, confirming the aryl halide activation as the rate determining step and that this step precedes nucleophile addition. The exact nature of the aryl halide activation step however has been a matter of some debate in the literature. Historically, at least with first generation ligands, consensus had come to favour oxidative addition of the aryl halide to a ligated copper(i) centre, thus generating an active copper(iii) intermediate.^14–23^ However, some recent studies have challenged this assumption: Hartwig and co-workers have shown that for their use of oxalohydrazido ligands the aryl halide activation likely occurs at a Cu(ii) centre to form a Cu(iii) intermediate with additional radical character supported on the ligand,^13,24^ and Shen *et al.* have suggested a multistep Cu(i)/Cu(iii)/Cu(ii)/Cu(iii)/Cu(i) redox sequence to be operative for related Ullmann-type coupling (C–CF_3_ bond formation).^29^

Addition of one equivalent of the radical inhibitor TEMPO to the model reaction (Scheme 2) caused a marked reduction in reaction rate and significantly lowered the yield of the cross-coupled product (see SI). This behaviour is inconsistent with expectations for a Cu(i)–Cu(iii) cycle^30^ and also contrasts with Shen's proposed mechanism.^29^ A second diagnostic experiment using 0.005 mol% Cu^II^Br_2_ as the pre-catalyst in place of Cu^I^I, afforded product 3 in 96% yield (TON = 1.9 × 10^4^). The comparable performance of Cu(i) and Cu(ii) precursors aligns with Hartwig's observations that Cu(ii) is the catalytically relevant oxidation state.^24^

**Scheme 3:**
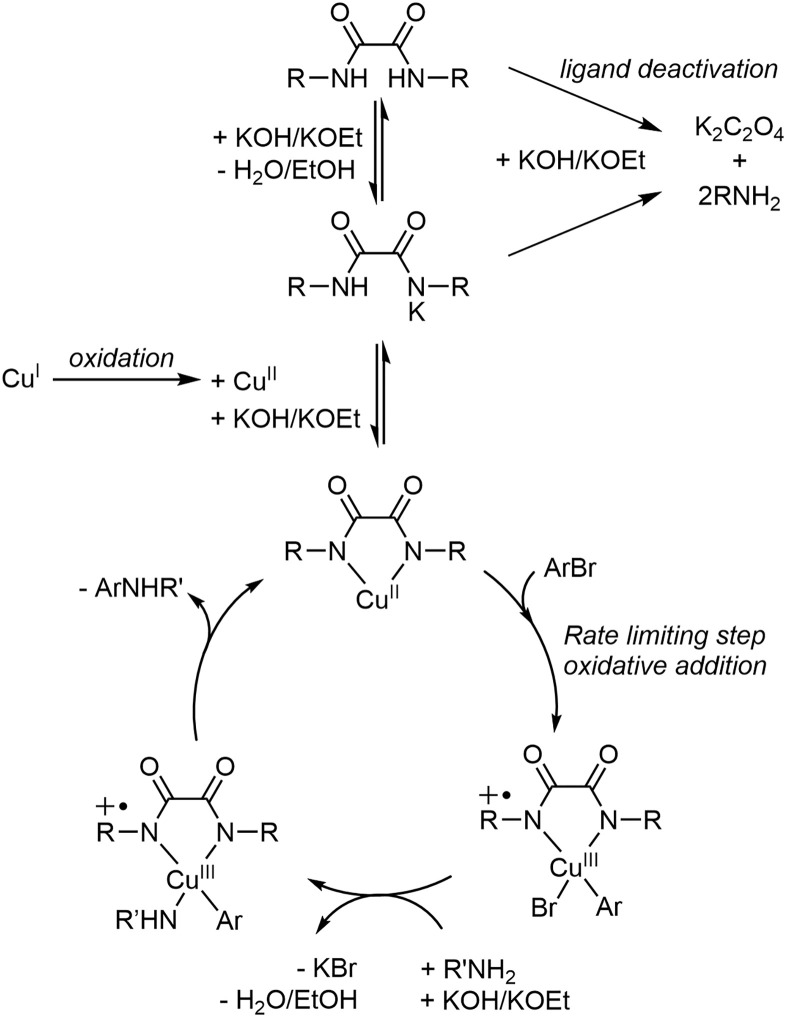
Potential mechanism of the cross-coupling reaction including ligand decomposition pathway.

The complexity of these systems, the lability of Cu(i)/(ii) complexes, and the presence of off-cycle equilibria make definitive assignment of the catalytic cycle challenging. Nevertheless a mechanistic proposal consistent with the present data and with prior literature (notably Hartwig's recent studies)^13,31^ is outlined in Scheme 3.

The oxalamide ligand is expected, based on its p*K*_a_ values, to exist in equilibrium with its mono-deprotonated form, the latter being dominant under the reaction conditions. Both species are susceptible to irreversible ligand hydrolysis deactivation as shown. Coordination of the mono-anion to copper would lower the p*K*_a_ of the remaining N–H proton, promoting full deprotonation and formation of the active copper complex. In this species, copper is present as Cu(ii), consistent with recent findings that Cu(i) can be rapidly oxidised to Cu(ii) by aryl halides.^13,31^ Oxidation by dioxygen present in the vial headspace is also plausible.

Oxidative addition of the aryl bromide to the copper(ii) complex would then constitute the rate limiting step and would generate a Cu(iii) intermediate with a radical stabilized on the oxalamide ligand.^13^ Subsequent amine coordination and deprotonation, followed by reductive elimination would furnish the cross-coupled product and regenerate the Cu(ii) catalyst. The base is depicted as KOH/KOEt with KOEt expected to be the major species due to the excess of EtOH.

## Conclusions

A fully homogeneous protocol for copper-catalyzed amination of aryl bromides employing oxalamide ligands has been developed, through targeted refinements of Ma's original methodology.^11^ This advancement has enabled us to undertake kinetic profiling and mechanistic interrogation under well-defined reaction conditions using second generation oxalamide ligands. The data obtained mainly aligns with prior studies using first generation ligands, confirming the aryl halide activation as the rate determining step and that this step precedes nucleophile addition. A plausible mechanism for the catalytic cycle is proposed which proceeds *via* a Cu(ii) initiated pathway similar to that previously reported by the Hartwig group.^13^

However, the most significant findings in terms of advancing catalytic performance concern catalyst decomposition pathways and their role in the reaction system. Our results reveal that the copper center exhibits remarkable stability with no evidence of any copper deactivation under the reaction conditions (EtOH solvent and KOH base), thus enabling extremely low loadings of metal over extended reaction times. In contrast, the oxalamide ligand undergoes relatively rapid base hydrolysis, which necessitates the use of an excess of ligand to maintain catalytic activity. These findings therefore diverge from the majority of reported Ullmann methodologies, which utilise DMSO or DMF in combination with milder bases like K_3_PO_4_ or Cs_2_CO_3_, conditions known to cause substantial copper deactivation yet mainly preserve oxalamide ligand integrity.

By compensating for ligand instability through controlled excess, we have shown how copper concentrations can be reduced to as little as 5 ppm (0.0005 mol%) for aryl iodides and 50 ppm (0.005 mol%) for aryl bromides while still delivering excellent yields. The turnover numbers for copper achieved under these conditions are, to our knowledge, the highest reported to date for Ullmann-type aminations, and rival or exceed those observed in related palladium-catalyzed couplings. The combination of low toxicity and minimal metal usage strengthens the case for copper as a greener alternative to palladium, particularly in pharmaceutical and agrochemical processes^7^ where residual metal levels in active pharmaceutical ingredients (API) are regulated under ICH Q3D guidelines.^32^ In addition, this work provides a foundation for further improvements in Cu-catalyzed bond-forming reactions, particularly for aryl chloride activation where catalyst loadings still remain high. Increasing the steric bulk of the oxalamide *N*-substituent enhances ligand stability but can negatively impact the rate-determining step, indicating that an optimal balance between steric protection and catalytic efficiency is required. Efforts to refine this balance are ongoing.

## Conflicts of interest

There are no conflicts to declare.

## Supplementary Material

RA-OLF-D6RA05376A-s001

## Data Availability

Full experimental data supporting this article, including all NMR spectra, have been included as part of the supplementary information (SI). Supplementary information is available. See DOI: https://doi.org/10.1039/d6ra05376a.

## References

[cit1] Vitaku E., Smith D. T., Njardarson J. T. (2014). J. Med. Chem..

[cit2] Buskes M. J., Blanco M. J. (2020). Molecules.

[cit3] Zhu Y., Dong W., Tang W. (2022). Adv. Agrochem.

[cit4] Ruiz-Castillo P., Buchwald S. L. (2016). Chem. Rev..

[cit5] Evano G., Blanchard N., Toumi M. (2008). Chem. Rev..

[cit6] Bhunia S., Pawar G. G., Kumar S. V., Jiang Y., Ma D. (2017). Angew. Chem., Int. Ed. Engl..

[cit7] Yang Q., Zhao Y., Ma D. (2022). Org. Process Res. Dev..

[cit8] Cai Q., Zhou W. (2020). Chin. J. Chem..

[cit9] Larsson P. F., Correa A., Carril M., Norrby P. O., Bolm C. (2009). Angew. Chem., Int. Ed. Engl..

[cit10] Buchwald S. L., Bolm C. (2009). Angew. Chem., Int. Ed. Engl..

[cit11] Gao J., Bhunia S., Wang K., Gan L., Xia S., Ma D. (2017). Org. Lett..

[cit12] Ray R., Hartwig J. F. (2021). Angew. Chem., Int. Ed. Engl..

[cit13] Pierson C. N., Horak T., Amberg W. M., Ray R., Rao G., Pinkhassik T. M., Fantasia S. M., Rummelt S. M., Puntener K., Britt R. D., Hartwig J. F. (2025). J. Am. Chem. Soc..

[cit14] Lo Q. A., Sale D., Braddock D. C., Davies R. P. (2019). Eur. J. Org Chem..

[cit15] Lo Q. A., Sale D., Braddock D. C., Davies R. P. (2017). ACS Catal..

[cit16] Sung S., Braddock D. C., Armstrong A., Brennan C., Sale D., White A. J., Davies R. P. (2015). Chemistry.

[cit17] Sung S., Sale D., Braddock D. C., Armstrong A., Brennan C., Davies R. P. (2016). ACS Catal..

[cit18] Ma X., Davies R. P. (2022). Adv. Synth. Catal..

[cit19] Jin X., Lin Y., Davies R. P. (2023). Catal. Sci. Technol..

[cit20] Jin X., Nguyen B. T., Davies R. P. (2024). Dalton Trans..

[cit21] Sperotto E., van Klink G. P. M., van Koten G., de Vries J. G. (2010). Dalton Trans..

[cit22] Sambiagio C., Marsden S. P., Blacker A. J., McGowan P. C. (2014). Chem. Soc. Rev..

[cit23] Gombert D. A., Darù A., Ahmed S. T., Haibach C. M., Li-Matsuura R., Yang C., Henry F. R., Cook P. S., Shekhar S., Blackmond G. D. (2023). ACS Catal..

[cit24] Delaney C. P., Lin E., Huang Q., Yu I. F., Rao G., Tao L., Jed A., Fantasia S. M., Puntener K. A., Britt R. D., Hartwig J. F. (2023). Science.

[cit25] Blackmond D. G. (2005). Angew. Chem., Int. Ed. Engl..

[cit26] Nielsen C. D., Bures J. (2019). Chem. Sci..

[cit27] Bhunia S., Kumar S. V., Ma D. (2017). J. Org. Chem..

[cit28] Zhou W., Fan M., Yin J., Jiang Y., Ma D. (2015). J. Am. Chem. Soc..

[cit29] Luo Y., Li Y., Wu B., Wang G., Wu J., Zhang S. Y., Houk K. N., Shen Q. (2025). Nature.

[cit30] Tseng C. K., Lee C. R., Tseng M. C., Han C. C., Shyu S. G. (2014). Dalton Trans..

[cit31] Zhao W., Amberg W. M., Rao G., Xie Y., Pierson C. N., Fantasia S. M., Rummelt S. M., Puntener K., Britt R. D., Hartwig J. F. (2026). J. Am. Chem. Soc..

[cit32] Q3D(R2) (Guideline for Metal Impurities), ICH, April 26, 2022, https://database.ich.org/sites/default/files/Q3D-R2_Guideline_Step4_2022_0308.pdf. Accessed Jan 9, 2025

