# Supplementary material for: High-turnover copper-catalyzed amination of aryl bromides: exploring catalyst and ligand degradation pathways
Source: RSC Adv. 2026 Jul 3;16(35):36665–72. doi: 10.1039/d6ra05376a (PMC13331200; doi:10.1039/d6ra05376a)
Supplement: RA-016-D6RA05376A-s001 [file RA-016-D6RA05376A-s001.pdf]

Supplementary Information (SI) for RSC Advances.
This journal is © The Royal Society of Chemistry 2026

Supporting Information

High-Turnover Copper-Catalyzed Amination of Aryl Bromides: Exploring Catalyst and Ligand Degradation Pathways

Tania Di Felice,[a] Troy Yu-Ting Chen,[a] David Sale,[b] D. Christopher Braddock,[a] Robert P Davies*[a]

[a] *Department of Chemistry, Molecular Sciences Research Hub, Imperial College London, White City Campus, 82 Wood Lane, London, W12 0BZ, UK.*

[b] Process Studies Group, Jealott's Hill Research Centre, Syngenta, Bracknell, Berkshire RG42 6EY, United Kingdom.

**Corresponding Author*

E-mail: r.davies@imperial.ac.uk

Tel: +44 (0)207 5945754

Contents

1 General Information 3
2 Experimental Studies 3
2.1 Synthesis of Oxalamide Ligand **MNBO** 3
2.2 Optimisation for Kinetic Studies 4
2.2.1 General Procedure of Optimisation for Kinetic Studies 4
2.2.2 Saturation Points of Solids 4
2.2.3 Reaction Side-Products 4
2.2.4 Reaction By-Products 5
2.2.5 Quantitative NMR Analysis 6
2.3 Kinetic Studies 7
2.3.1 General Procedure for Kinetic Studies 7
2.3.2 Reproducibility of Reactions for Kinetic Studies 7
2.3.3 Temperature Profile of Reactions at Reflux in EtOH 10
2.3.4 Addition of TEMPO 11
2.4 Ligand Degradation Studies 11
2.4.1 Synthesis of Oxalamide Ligands **L1**-**L4** 11
2.4.2 Ligand Degradation Profiles 13
2.5 Reaction Scope Studies 14
2.5.1 General Procedure 14
2.5.2 Scale-up Synthesis of **3a** 14
2.5.3 Reaction Scope Products 15
3 NMR Spectra 21
3.1 ^{1}H and ^{13}C Spectra of Ligands 21
3.2 ^{1}H Spectra for Reaction Scope Products 26
4 References 36

1 General Information

All glassware were dried overnight in a 120 °C oven before use. All reagents were purchased commercially and were used as received. Stock solutions of $Cu(MeCN)_4.OTf$ and **MNBO** were prepared in glass vials with septum lids and heated to reaction temperature prior to addition. Optimisation and scope reactions and the ligand degradation experiments were performed in sealed vials under ambient atmosphere for operational simplicity. Kinetic experiments were conducted using quickfit laboratory glassware under a nitrogen atmosphere to facilitate easier sampling over the course of the reaction.

All yields reported were determined by ^1H NMR, where naphthalene or 1,3,5-trimethoxybenzene was used as an internal standard. ^1H, ^{13}C and ^{19}F NMR spectroscopy data were obtained at room temperature (unless otherwise specified) using Bruker AV-400 spectrometers. Chemical shifts δ are reported in parts per million (ppm) from tetramethylsilane (TMS) and are referenced to the residual ^1H and ^{13}C signal of deuterated solvents. Coupling constants (*J*) are given in Hz units. All spectra were analysed using MESTRELAB MestReNova software.

GC-MS analysis was carried out on a Micromass Autospec Premier Spectrometer using the following conditions: 50 °C held for 5 mins, then 10 °C/min to 290 °C, flow rate of 1 mL/min He, DB5 column with dimensions 30 m x 2.5 mm x 0.25 µm, EtOAc solvent. ESI-MS analysis was carried out on a Waters LCT Premier Spectrometer.

2 Experimental Studies

2.1 Synthesis of Oxalamide Ligand MNBO

MNBO was synthesised according to the procedure reported by Ma *et. al.*[1] To a 250 mL round bottom flask under nitrogen, mono-methyloxalyl chloride (3.0 mL, 33 mmol, 1.1 eq.) was added dropwise to a mixture of 2-methylnaphthalenylamine (2.8 mL, 30 mmol, 1.0 eq.), Et_3N (5.0 mL, 36 mmol, 1.2 eq.) and THF (60 mL) at 0 °C. The mixture was stirred at room temperature for 2 hours. Water (30 mL) and EtOAc (3 x 10 mL) were added, and the combined organics were dried over $MgSO_4$. The solvent was removed via rotary evaporation to yield an orange viscous oil. The crude product was purified with silica gel chromatography using n-hexane:EtOAc (10:0 → 8:2) to afford a pale-yellow viscous oil (4.3 g, 18 mmol, 59 %). The product was dissolved in THF (20 mL), and $BnNH_2$ (2.3 mL, 21 mmol, 1.2 eq.) was added at room temperature. The mixture was then heated to 70 °C and stirred for 1 hour. The mixture was cooled to room temperature, then to 0 °C. n-Hexane (~5 mL) was added to induce precipitation. The solid product was obtained via suction filtration and washed with cold diethyl ether (15 mL) to afford **MNBO** as a white powder (4.2 g, 13 mmol, 40 %). Mp.: 190 °C. ^1H NMR (400 MHz, $CDCl_3$) δ 9.27 (s, 1H, NH), 7.99 - 7.90 (m, 1H, Ar, NH), 7.90 - 7.83 (m, 2H, Ar), 7.83 - 7.78 (d, 1H, *J* = 8.4Hz, Ar), 7.60 - 7.45 (m, 2H, Ar), 7.45 - 7.32 (m, 6H, Ar), 4.66 - 4.59 (d, 2H, *J* = 6.28 Hz, CH_2), 2.44 (s, 3H, CH_3). ^{13}C NMR (100 MHz, $CDCl_3$) δ 159.7 (C=O), 158.5 (C=O), 136.6 (Ar), 133.0 (Ar), 132.7 (Ar), 129.9 (Ar) , 128.9 (Ar) , 128.8 (Ar) , 128.3 (Ar) , 128.2 (Ar) , 128.2 (Ar), 128.1 (Ar) , 128.0 (Ar), 126.9 (Ar), 125.6 (Ar), 122.0 (Ar), 44.1 (CH_2), 18.8 (CH_3). HRMS (ES+, TOF): *m/z* 319.1447 $[M+H]^+$, calculated for $[C_{20}H_{19}N_2O_2]^+$: 319.1447.

2.2 Optimisation for Kinetic Studies

2.2.1 General Procedure of Optimisation for Kinetic Studies

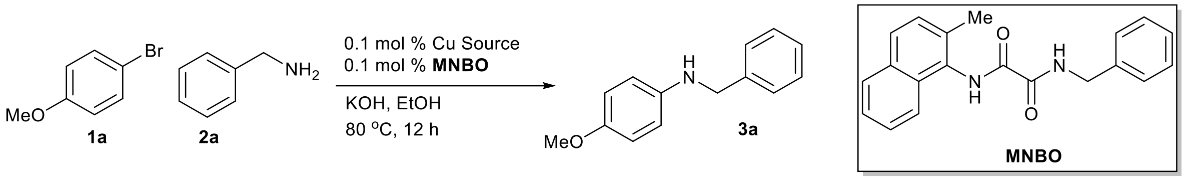

Scheme S1 General scheme for optimisation of reaction.

4-Bromoanisole (1.252 mL, 10.00 mmol, 1 eq.), benzylamine (1.643 mL, 15.00 mmol, 1.5 eq.), copper source (Cu_2O: 1.4 mg, $Cu(MeCN)_4$.OTf: 3.8 mg, $CuBr_2$: 2.2 mg, 0.010 mmol. 0.001 eq.), **MNBO** (3.2 mg, 0.010 mmol, 0.001 eq.), KOH (0.7294 g, 13.00 mmol, 1.3 eq.), EtOH (1 or 2 mL) and 1,3,5-trimethoxybenzene (0.8410 g, 5.000 mmol) or naphthalene (0.6405 g, 5.000 mmol) were placed in a 14 mL glass vial. The mixture was stirred and heated at 80 °C for 12 hours, then cooled to room temperature and diluted with ethyl acetate (7 mL). The mixture was washed with brine (2 x 7 mL) and the organic phase was dried over $MgSO_4$. The solvent was removed by rotary evaporation. An NMR was taken of the crude product in $CDCl_3$, and the yield calculated by quantitative NMR with 1,3,5-trimethoxybenzene or naphthalene as the internal standard.

2.2.2 Saturation Points of Solids

The approximate saturation points of KOH and **MNBO** were determined under reaction conditions (EtOH solvent, 80 °C) so that homogeneity can be fully realised for future reactions when altering the concentrations. $Cu(MeCN)_4$.OTf is highly soluble in hot EtOH. The approximate saturation point of **MNBO** can be increased to 0.05 M when in the presence of $Cu(MeCN)_4$.OTf.

Entry	Solid Reagent	Concentration (M)
1	KOH	7.2
2	**MNBO**	0.025

Table S1 Saturation points of solid reagents in EtOH at 80 °C.

2.2.3 Reaction Side-Products

The side-products for the reaction shown in Scheme S1 in EtOH were confirmed by GC-MS (Figure S1) and ^{1}H NMR. The side product 4-methoxyphenol (**4**) is likely formed from reaction with H_2O / KOH which itself is generated in-situ as a by-product of the reaction. Upon addition of an excess of H_2O to the reaction mixture, the yield of **4** was seen to increase (Table S2). **5** has been previously reported in literature to form due to the in-situ formation of potassium ethoxide.[2]

Entry	Additive	NMR Yield of 3a (%)	NMR Yield of 4 (%)
1	-	93	2
2	H_2O (28 mmol)	42	52
3	Molecular Sieves (0.5 g, 4 Å)	97	0

*Table S2 **S1** forms from water formed in-situ during the reaction.*

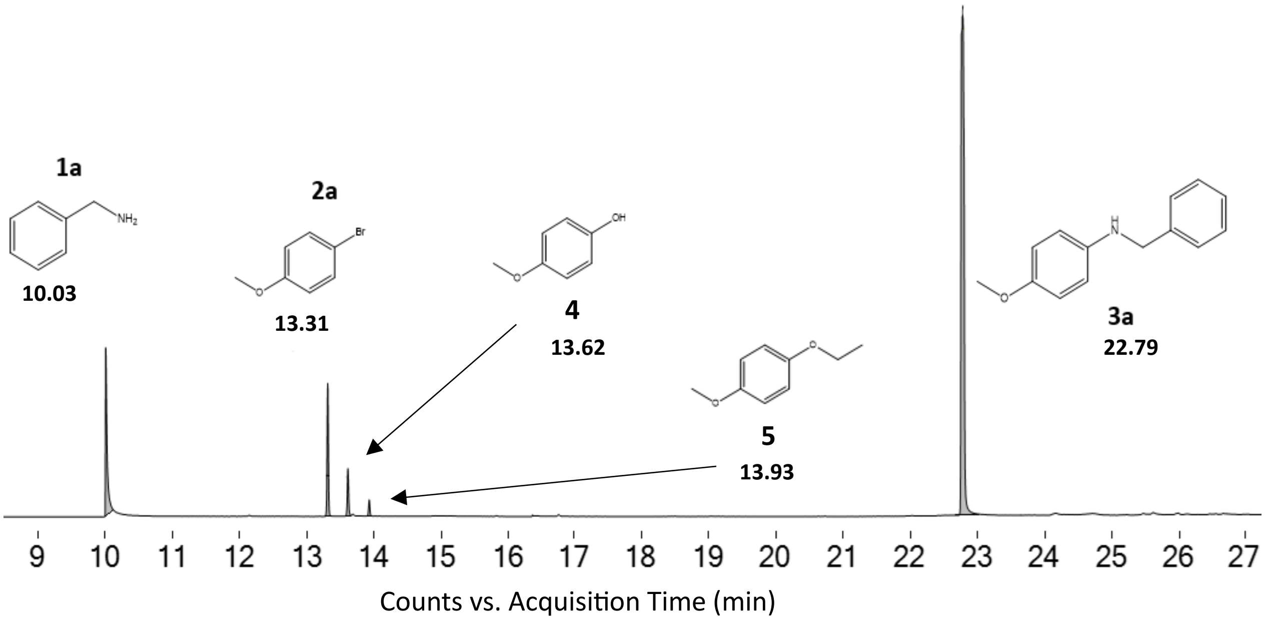

Figure S1 GC-MS spectrum of reaction mixture containing starting materials, major product and side-products.

2.2.4 Reaction By-Products

A white precipitate forms during the reaction. The powder X-ray diffraction (pXRD) data of this solid (Figure S2, left) matches very well with the literature data for KBr (Figure S2, right).

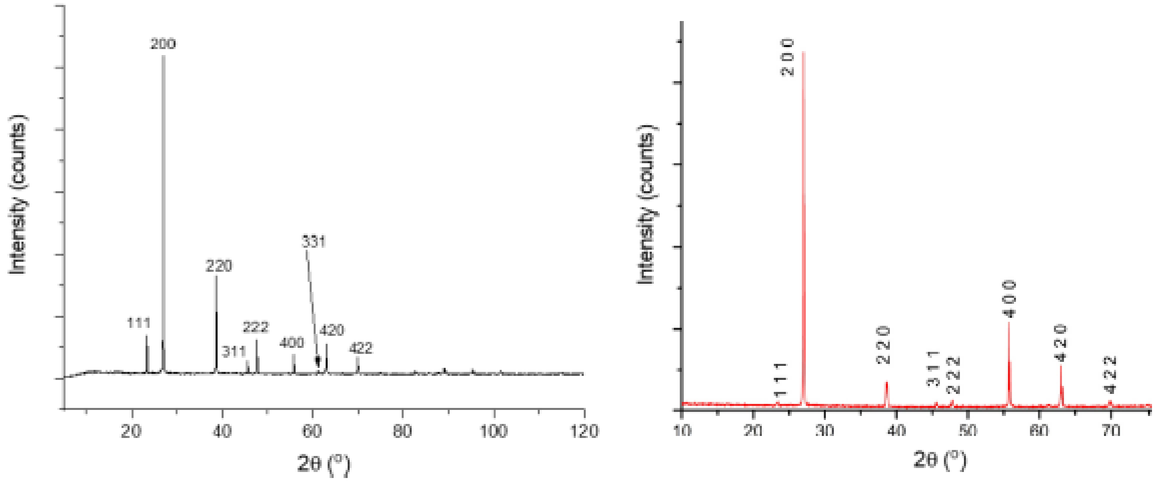

Figure S2 Left: pXRD of white precipitate from reaction. Right: Literature data for KBr.[3]

2.2.5 Quantitative NMR Analysis

Amine **2a** is miscible in water so some may be lost during the work-up, therefore its concentration cannot be accurately determined by NMR. The concentration of **1a** and **3a** have been determined by quantitative NMR against naphthalene as an internal standard. Naphthalene was chosen as a suitable internal standard since it does not participate in the reaction, the ^{1}H NMR peaks do not overlay with any others in the reaction mixture and the reference protons have a similar relaxation time (T_1 = 4.3 – 4.9 s) to the OMe group in **1a** (T_1 = 5.3 s) and the CH_2 peak in **3a** (T_1 = 4.7 s) which are the peaks used to quantify the concentrations of **1a** and **3a**.

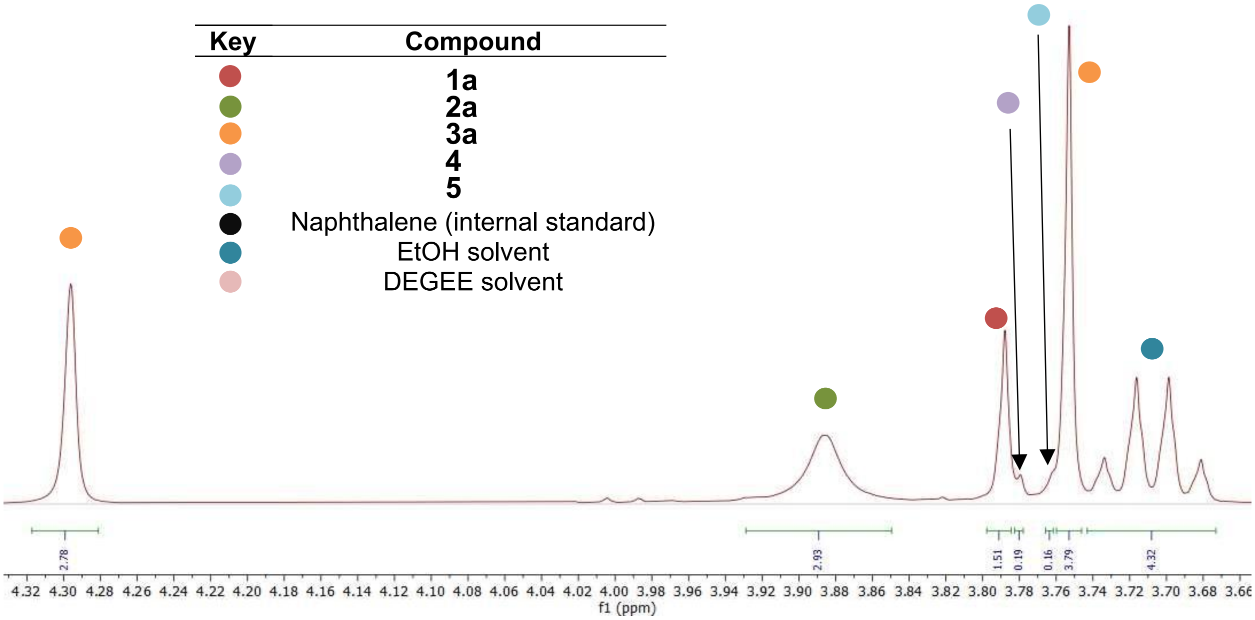

*Figure S3 NMR spectrum of reaction mixture in EtOH. The peak of **3a** at 4.3 ppm is used for product quantification.*

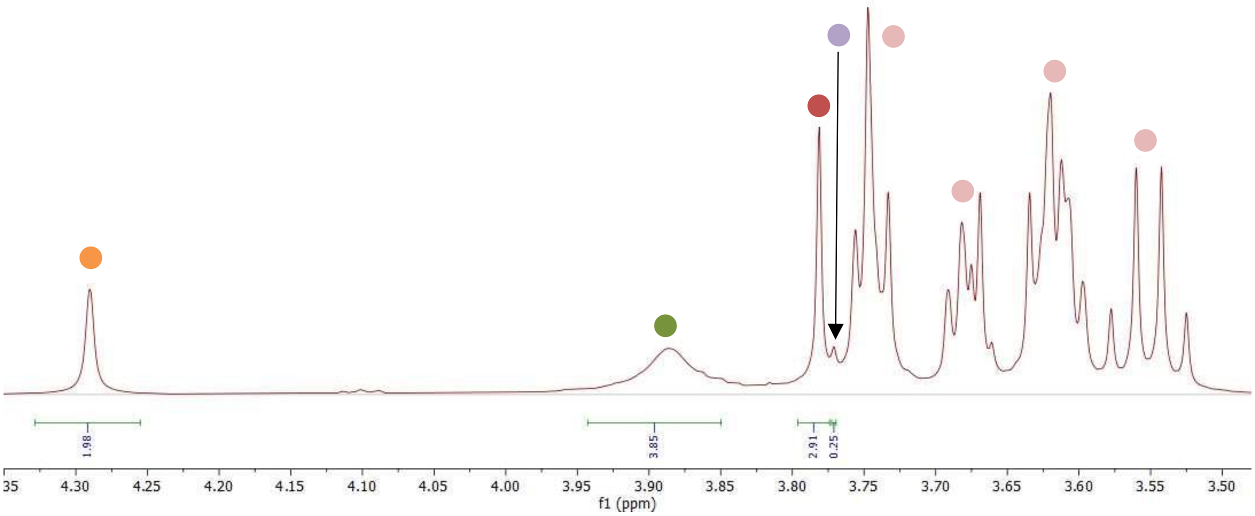

Figure S4 NMR spectrum of reaction mixture in DEGEE (see Key in Figure S3).

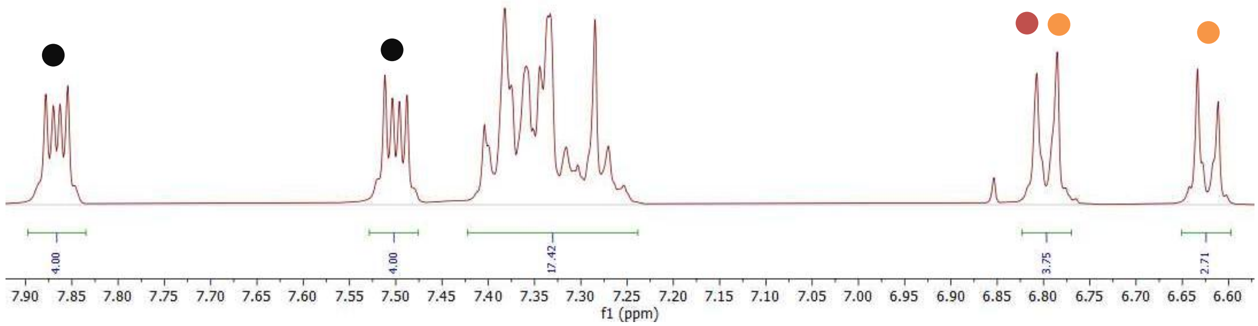

Figure. S5 NMR spectrum of aromatic region of reaction mixture DEGEE (see Key in Figure S3).

2.3 Kinetic Studies

2.3.1 General Procedure for Kinetic Studies

Representative examples of the general procedure for the standard reactions in EtOH and DEGEE are reported below.

EtOH: To a 3-neck 100 mL round bottom flask fitted with a condenser and mercury thermometer, naphthalene (3.203 g, 25 mmol), KOH (3.647 g, 65 mmol), EtOH (13.5 mL), benzylamine (8.192 mL, 75 mmol) and 4-bromoanisole (6.276 mL, 50 mmol) were added and heated to reflux (112 °C). The mixture was stirred for 20 minutes until the solids were dissolved. $Cu(MeCN)_4$.OTf and **MNBO** were added via stock solution (0.05 M in ethanol, 1 mL) to initiate the reaction. ~0.1 mL aliquots were collected over the course of the reaction (6 h) and were placed in a screw-cap vial with brine (0.6 mL) and $CDCl_3$ (0.6 mL). The aqueous layer was washed with further $CDCl_3$ (0.6 mL). The combined organic layers were dried over $MgSO_4$ and filtered into an NMR tube.

DEGEE: To a 3-neck 100 mL round bottom flask fitted with a condenser and mercury thermometer, naphthalene (3.203 g, 25 mmol), KOH (3.647 g, 65 mmol), DEGEE (13.5 mL), benzylamine (8.192 mL, 75 mmol) and 4-bromoanisole (6.276 mL, 50 mmol) were added and heated to 105 °C. The mixture was stirred for 20 minutes until the solids were dissolved. $Cu(MeCN)_4$.OTf and **MNBO** were added via stock solution (0.05 M, 1 mL) to initiate the reaction. ~0.1 mL aliquots were collected over the course of the reaction (4 h) and were placed in a screw-cap vial with brine (0.6 mL) and $CDCl_3$ (0.6 mL). The aqueous layer was washed with further $CDCl_3$ (0.6 mL). The combined organic layers were dried over $MgSO_4$ and filtered into an NMR tube.

For all kinetic reactions, the total volume was kept at 29 mL by adjusting the volume of solvent used.

2.3.2 Reproducibility of Reactions for Kinetic Studies

The reactions were run several times under standard conditions with both EtOH and DEGEE solvents (Figure S6 and S7 respectively). The reaction traces showed excellent reproducibility both for consumption of **1a** and formation of **3a**. The traces labelled 'Reproducibility Test 1' in each solvent have been used as the standard reaction in subsequent analysis.

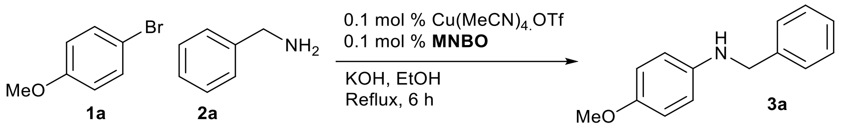

Symbol	[1a]$_0$ (M)	[2a]$_0$ (M)	[Cu(MeCN)$_4$.OTf]$_0$ (M)	[MNBO]$_0$ (M)	[KOH]$_0$ (M)	Solvent
● ● ●	1.7	2.6	0.002	0.002	2.2	EtOH
● ● ●	1.7	2.6	0.002	0.002	2.2	DEGEE

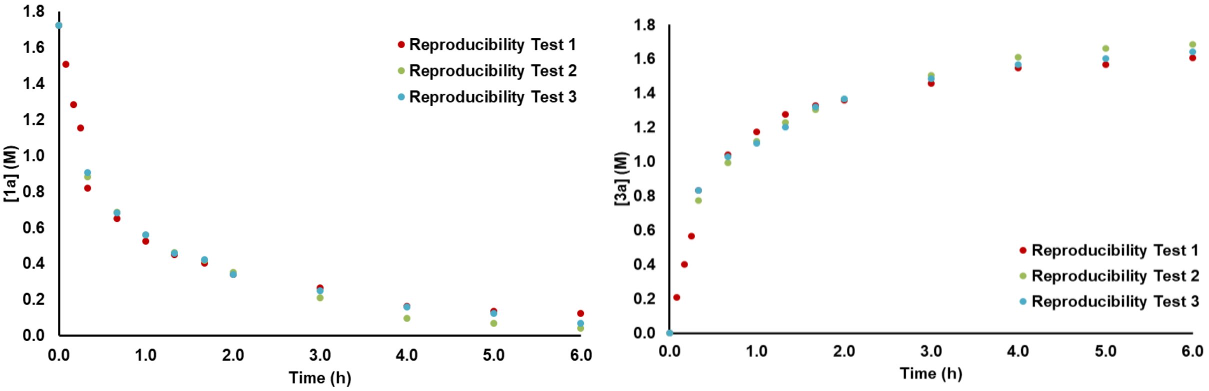

Figure S6 Reaction profiles for standard reaction using given conditions in EtOH.

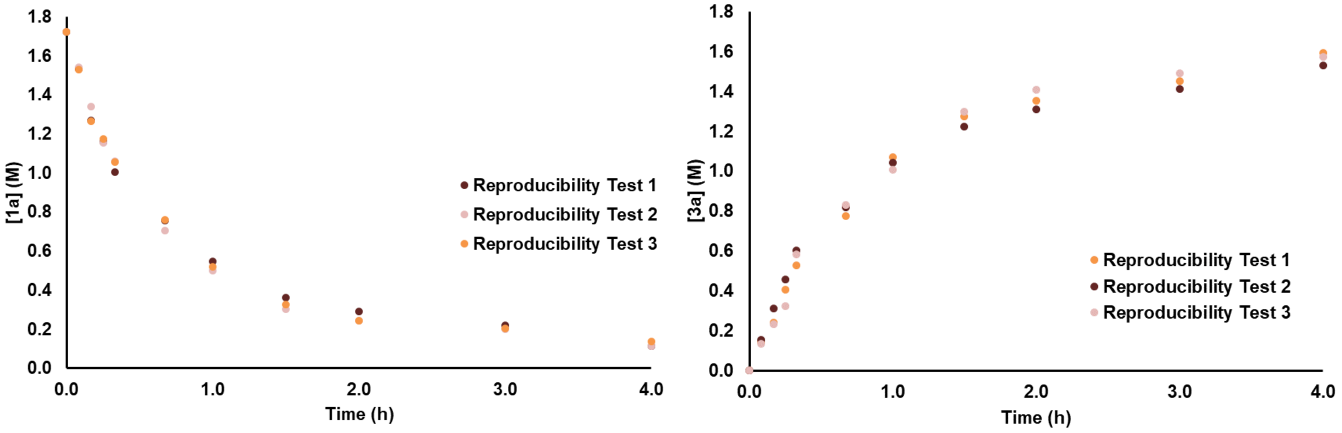

Figure S7 Reaction profiles for standard reaction using given conditions in DEGEE (see Key in Figure S6).

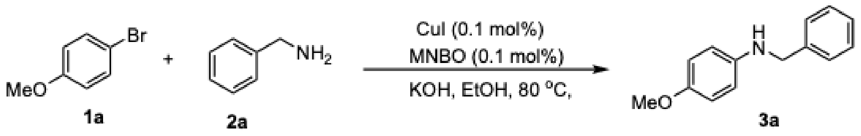

Symbol	$[1a]_0$ (M)	$[2a]_0$ (M)	$[CuI]_0$ (M)	$[MNBO]_0$ (M)	$[KOH]_0$ (M)
● ● ●	2.0	3.0	0.002	0.002	2.7

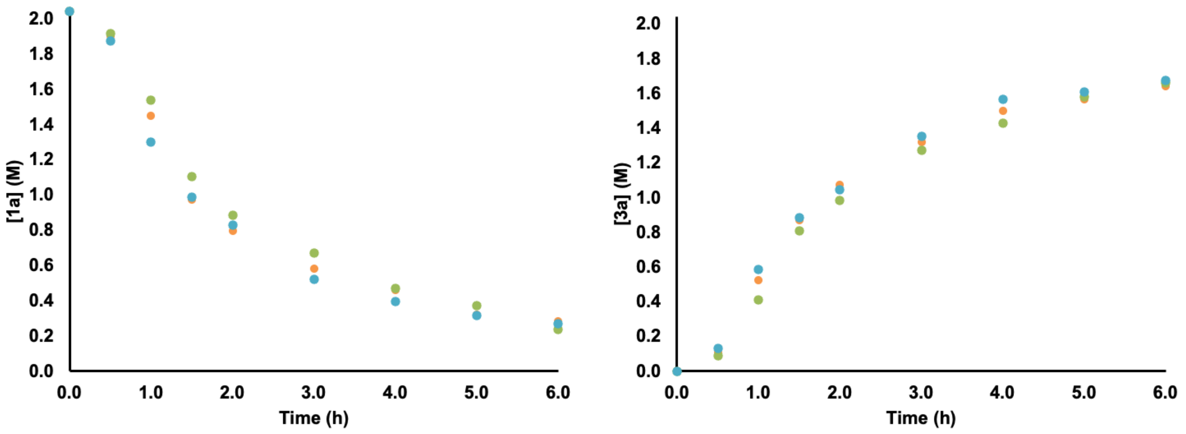

Figure S8 Reaction profiles using CuI (0.05M stock solution in MeCN) pre-catalyst.

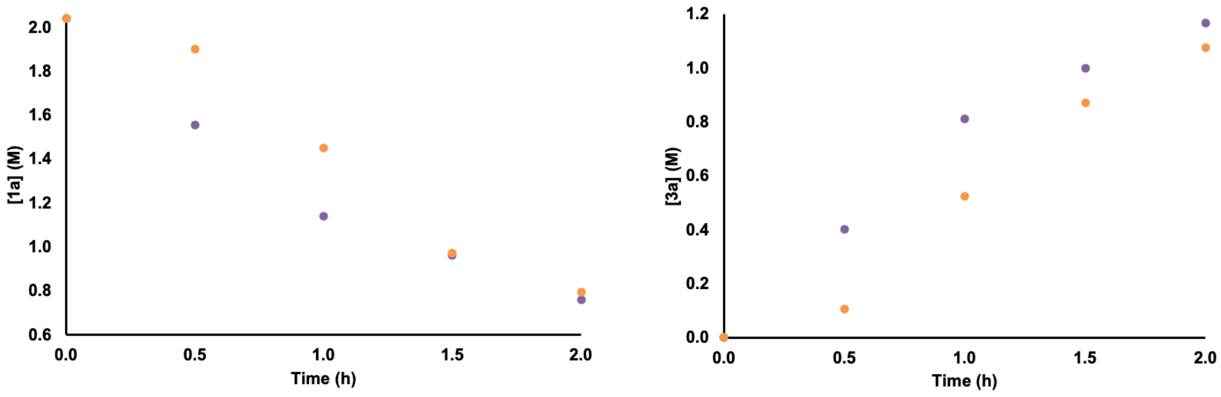

Figure S9 Reaction profiles over initial 2h for CuI administered as solid (purple) vs 0.05M stock solution in MeCN (orange), using reaction conditions shown in Figure S8.

2.3.3 Temperature Profile of Reactions at Reflux in EtOH

Due to the concentrated nature of the reaction mixture, and the use of higher boiling point starting materials (**1a**, 223 °C; **2a**, 185 °C), when the reaction was performed at reflux in EtOH, the initial boiling point of the mixture was higher than the boiling point of the solvent used; 112 °C compared to an expected reflux temperature of 78 °C. In addition, as the reaction proceeded and the starting materials were consumed, the reflux temperature of the mixture decreased. The reflux temperature stabilised at around 90 °C after 2 hours when > 70 % of **1a** had been consumed, therefore displaying a temperature range of 22 °C over the course of the reaction. Under same excess conditions, where the concentration of starting materials was decreased by more than a half, the reflux temperature was much lower at 88 °C and dropped to 83 °C over the course of the reaction. The changing temperature over the course of the reaction profile make it challenging to directly compare reactions carried out using different concentrations of reagents when using EtOH as solvent, hence leading to our use of DEGEE solvent for different excess experiments as discussed in the manuscript.

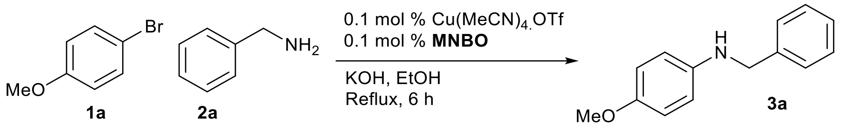

Symbol	$[1a]_0$ (M)	$[2a]_0$ (M)	$[Cu(MeCN)_4.OTf]_0$ (M)	$[MNBO]_0$ (M)	$[KOH]_0$ (M)
🔴	1.7	2.6	0.002	0.002	2.2
🟢	0.7	1.6	0.002	0.002	1.2

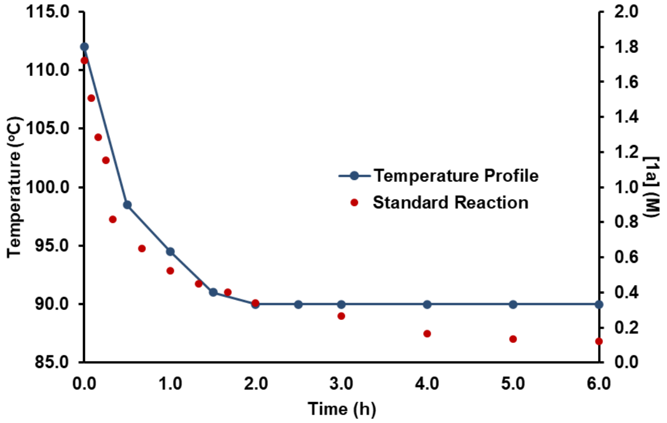

Figure S10 Reflux temperature of standard reaction in EtOH over the course of the reaction.

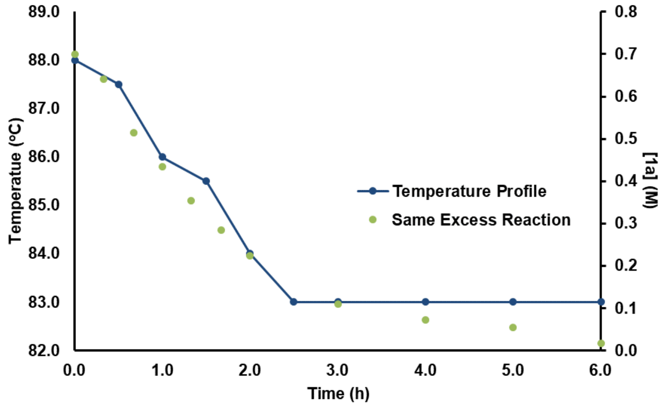

Figure S11 Reflux temperature of same excess reaction in EtOH over the course of the reaction.

2.3.4 Addition of TEMPO

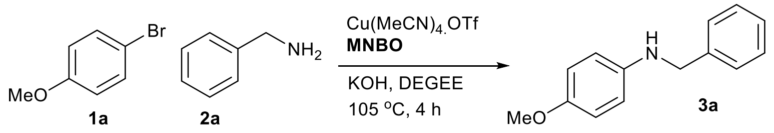

Symbol	$[1a]_0$ (M)	$[2a]_0$ (M)	$[Cu(MeCN)_4.OTf]_0$ (M)	$[MNBO]_0$ (M)	$[KOH]_0$ (M)	TEMPO (M)
●	2.0	3.0	0.002	0.002	2.7	0
●	2.0	3.0	0.002	0.001	2.7	2.0

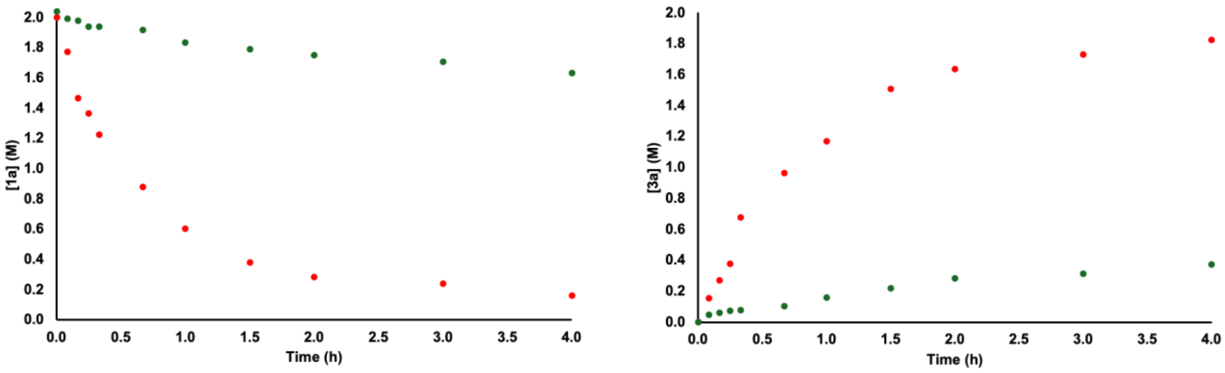

Figure S12 Concentration profiles of reactions with an without addition of TEMPO

2.4 Ligand Degradation Studies

2.4.1 Synthesis of Oxalamide Ligands **L1**-**L4**

*2.4.1.1 N,N'-bis(2-methylnaphthalen-1-yl)oxalamide (**L1**).*

To a solution of 2-methyl-1-naphthylamine (1.8 mL, 20 mmol, 2.0 equiv.), triethylamine (2.8 mL, 22 mmol, 2.1 equiv.), and THF (40 mL) in an ice bath, oxalyl chloride (0.8 mL, 10 mmol, 1.0 equiv.) was added dropwise. The mixture was then stirred at room temperature for 2 hours. After the reaction, the solvent was evaporated under reduced pressure, yielding a yellow solid. This solid was treated with water (10 mL), filtered and washed with water (3 × 5 mL) and cold diethyl ether (6 mL). The product was dried in a vacuum oven at 40°C overnight. Purification by recrystallization from ethyl acetate afforded **L1** as a pale-yellow solid (3.13 g, 8.5 mmol, 85%). ^{1}H NMR (400 MHz, d6-DMSO) δ 10.84 (s, 2H), 7.95 (d, 2H, J = 8 Hz), 7.89 (d, 2H, J = 8 Hz), 7.62 (d, 2H, J = 8 Hz), 7.59 (t, 2H, J = 4 Hz), 7.55 (t, 2H, J = 4 Hz), 7.52 (d, 2H, J = 8 Hz), 2.43 (s, 6H). ^{13}C NMR (101 MHz, d6-DMSO) δ 160.2, 133.1, 130.7, 129.1, 127.7, 128.9, 126.9, 125.8, 123.5, 18.7.

This compound has been previously reported.[1]

2.4.1.2 N,N'-dibenzyloxalamide (L2)

To a solution of benzylamine (2.2 mL, 20 mmol, 2.0 equiv.), triethylamine (2.8 mL, 22 mmol, 2.1 equiv.), and THF (40 mL) in an ice bath, oxalyl chloride (0.8 mL, 10 mmol, 1.0 equiv.) was added dropwise. The mixture was then stirred at room temperature for 2 hours. After the reaction, the solvent was evaporated under reduced pressure, yielding a white solid. This solid was treated with water (10 mL), filtered, and washed with water (3 × 5 mL) and cold diethyl ether (6 mL). The product was dried in a vacuum oven at 40°C overnight. Purification by recrystallization from ethyl acetate afforded **L2** as a white solid (2.41 g, 9 mmol, 90%). ^{1}H NMR (400 MHz, $CDCl_3$) δ 7.80 (s, 2H), 7.35 (m, 10H), 4.53 (d, 4H). ^{13}C NMR (100 MHz, CDCl3) δ 159.6, 136.7, 128.9, 127.9, 127.8, 43.8.

This compound has been previously reported.[4]

2.4.1.3 N,N'-bis(4-methoxy-2,6-dimethylphenyl)oxalamide (**L3**)

To a solution of 4-amino-3,5-xylenol (2.7 g, 20 mmol, 2.0 equiv.), triethylamine (2.8 mL, 22 mmol, 2.1 equiv.), and THF (40 mL) in an ice bath, oxalyl chloride (0.8 mL, 10 mmol, 1.0 equiv.) was added dropwise. The mixture was then stirred at room temperature for 2 hours. After the reaction, the solvent was evaporated under reduced pressure, yielding a yellow solid. This solid was treated with water (10 mL), filtered, and washed with water (3 × 5 mL) and cold diethyl ether (6 mL) to give *N,N'*-bis(4-hydroxy-2,6-dimethylphenyl)oxalamide as the precursor. The product was dried in a vacuum oven at 40°C overnight without further purification. A solution of the resultant product in DMF (0.5 M, 20 mL) was combined with potassium carbonate (3.5 g, 25 mmol, 2.5 equiv.) and iodomethane (1.9 mL, 30 mmol, 3.0 equiv.) and stirred under a nitrogen atmosphere overnight. The crude reaction mixture was then quenched with water and the precipitate was collected by suction filtration. The solid residue was washed with an excess of water to remove all remaining DMF, followed by cold diethyl ether (6 mL). Purification by recrystallization from ethyl acetate afforded **L3** as a white solid (2.74 g, 7.7 mmol, 77%). ^{1}H NMR (400 MHz, d6-DMSO) δ 10.0 (s, 2H), 6.70 (s, 4H), 3.74 (s, 6H), 2.14 (s, 12H); ^{13}C NMR (101 MHz, d6-DMSO) δ 159.7, 158.1, 136.8, 53 127.5, 113.4, 55.6, 18.7.

This compound has been previously reported.[4]

2.4.1.4 N-benzyl-N'-(4-methoxy-2,6-dimethylphenyl)oxalamide (**L4**)

In a 100 mL round-bottom flask under nitrogen, methyl 2-chloro-2-oxoacetate (1.1 mL, 12 mmol, 1.2 equiv.) was added dropwise to a mixture of 4-amino-3,5-xylenol (1.4 g, 10 mmol, 1.0 equiv.), triethylamine (1.7 mL, 12 mmol, 1.2 equiv.), and THF (20 mL) at 0°C. The reaction was stirred at room temperature for 2 hours. Water (30 mL) was then added, and the mixture was extracted with ethyl acetate (3 × 15 mL). The combined organic layers were dried over magnesium sulfate, and the solvent was removed under vacuum, yielding the ester precursor as a yellow viscous oil without additional purification. The crude solid was then added into a solution of potassium carbonate (3.5 g, 25 mmol, 2.5 equiv.), DMF (0.5 M, 20 mL) and iodomethane (1.9 mL, 30 mmol, 3.0 equiv.) and stirred under a nitrogen atmosphere overnight. Water (30 mL) was then added, and the mixture was extracted with DCM (3 × 15 mL) and washed with brine and excess water (approx. 100mL) to remove all DMF. The combined organic layers were dried over magnesium sulfate, and the solvent was removed under vacuum, yielding the methylated ester precursor as a yellow viscous oil. The crude mixture was purified by silica gel chromatography with a gradient eluent of n-hexane and ethyl acetate (10:0 → 7:3), resulting in white solid with a yield of 67%. This pure product was dissolved in THF (20 mL), and benzylamine (1.3 mL, 12 mmol, 1.2 equiv.) was added at room temperature. The mixture was heated to 70°C and stirred for 1 hour. After cooling to room temperature, cold hexane (5-10 mL) was added to induce precipitation and the white precipitate formed instantaneously. The solid was collected by suction filtration and washed with cold diethyl ether (10 mL) and dried in a vacuum oven at 40°C overnight, resulting in **L4** as a white powder (1.41 g, 4.5 mmol, 45%). ^{1}H NMR (400 MHz, d6-DMSO) δ 10.0 (s, 1H), 9.42 (t, 1H), 7.31 (m, 5H),

6.68 (s, 2H), 4.38 (d, 2H), 3.73 (s, 3H), 2.09 (s, 6H); ^{13}C NMR (101 MHz, d6-DMSO) δ 160.6, 159.5, 158.2, 139.2, 136.8, 128.8, 128.0, 127.5, 113.4, 55.6, 43.0, 18.7.

This compound has been previously reported.[4]

2.4.2 Ligand Degradation Profiles

A 14 mL vial with a magnetic stir bar was loaded with oxalic diamide ligand (0.1 mmol), KOH (0.0729 g, 1.3 mmol, 13 equiv.) and ethanol (1.0 mL) and sealed. The reaction mixture was heated at 80 °C for a given time (1-48 hours) with vigorous stirring. Once the reaction was complete, it was quenched with water (2 mL) and extracted with ethyl acetate (3 × 5 mL) and DCM (3 × 5 mL). The combined organic layers were dried over anhydrous magnesium sulfate (approx. 0.5 g), and the solvent was removed under vacuum. Before performing ^{1}H NMR analysis, 1,3,5-trimethoxybenzene (0.017 g, 0.1 mmol) was added to the mixture as an internal standard.

For the degradation of **L2** it was shown that two 2-methyl-1-naphthylamine molecules were formed from each ligand, indicating complete hydrolysis of both amide groups in **L2**.

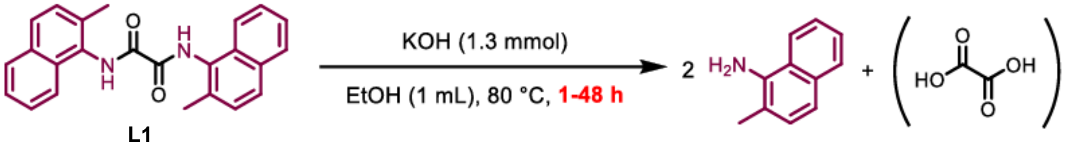

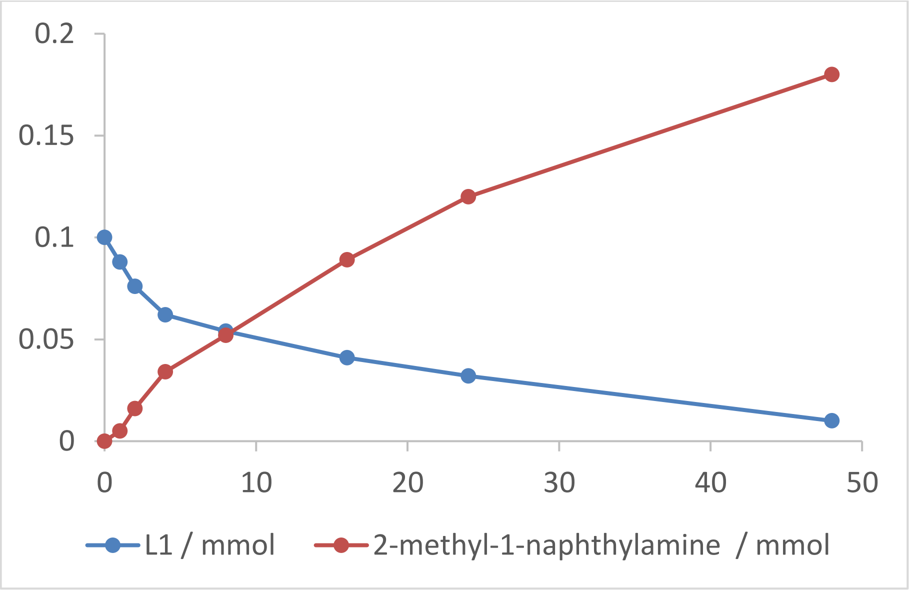

Figure S13. Degradation profile for **L1** (0.1 mmol ligand, 1.3 mmol KOH, 1 mL EtOH, 80 °C) as determined using ^{1}H NMR with 1,3,5-trimethoxybenzene as an internal standard.

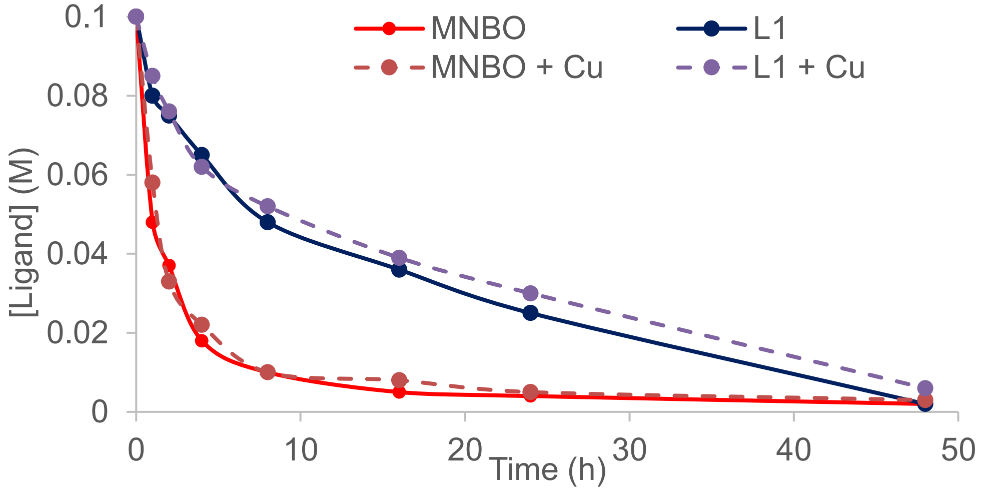

Figure S14. Degradation profile for MNBO and **L1** (0.1 mmol ligand, 1.3 mmol KOH, 1 mL EtOH, 80 °C) with and without addition of $Cu(MeCN)_4.OTf$ (5 x 10^{-4} mmol, added as 0.1 mL of a 0.0005 M stock solution in EtOH), as determined using ^1H NMR with 1,3,5-trimethoxybenzene as an internal standard.

2.5 Reaction Scope Studies

2.5.1 General Procedure

Aryl-bromide (5 mmol, 1 eq.), amine (7.5 mmol, 1.5 eq.), KOH (0.365 g, 6.5 mmol, 1.3 eq.), **MNBO** (1.6 mg, 0.005 mmol, 0.001 eq.), $Cu(MeCN)_4.OTf$ (2.5 x 10^{-4} mmol, added as 0.5 mL of a 0.0005 M stock solution in EtOH) and naphthalene (0.320 g, 2.5 mmol, 0.5 eq.) were added to a 7 mL screw-neck vial . The mixture was heated to 80 °C with magnetic stirring for 48 hours. Once complete the reaction was cooled to room temperature, EtOAc (3 mL) was added and then the solution was washed with a brine solution (3 x 3 mL). The organic phase was dried over $MgSO_4$ and the solvent removed on a rotary evaporator. An NMR was taken of the resulting crude product, and the yield calculated by qNMR using naphthalene as the internal standard.

2.5.2 Scale-up Synthesis of 3a

4-Bromoanisole (1.25 mL, 10.0 mmol, 1 eq), benzylamine (1.64 mL, 15.0 mmol, 1.5 eq), $Cu(MeCN)_4.OTf$ (5 x 10^{-4} mmol, added as 1.0 mL of a 0.0005 M stock solution in EtOH), **MNBO** (3.2 mg, 0.010 mmol, 0.001 eq.), KOH (0.729 g, 13.0 mmol, 1.3 eq.), and EtOH (2 mL) were placed in a 14 mL glass vial. The mixture was stirred and heated at 80 °C for 24 hours, then cooled to room temperature and diluted with ethyl acetate (7 mL). The mixture was washed with brine (2 x 7 mL) and the organic phase was dried over $MgSO_4$. The solvent was removed under vacuum using rotary evaporation to yield an orange oil which was purified with silica gel chromatography using n-hexane:EtOAc (19:1 → 9:1) to afford a pale yellow solid (1.855 g, 8.71 mmol, 87 %).

2.5.3 Reaction Scope Products

3a

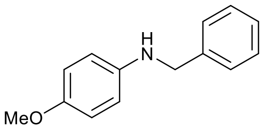

N-Benzyl-4-methoxyaniline (**3a**) was formed from 4-bromoanisole (0.626 mL, 5 mmol, 1 eq.) and benzylamine (0.822 mL, 7.5 mmol, 1.5 eq.) in 99 % yield.

^{1}H NMR (400 MHz, $CDCl_3$) δ 7.47 – 7.29 (m, 5H, Ar), 6.88 – 6.82 (dt, 2H, *J* = 8 Hz, Ar) 6.70 – 6.64 (dt, 2H, *J* = 8 Hz, Ar), 4.34 (s, 2H, CH_2), 3.93 (s, 1H, NH), 3.80 (s, 3H, CH_3).

This compound has been previously reported.[1]

3b

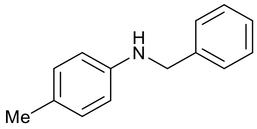

N-Benzyl-4-methylaniline (**3b**) was formed from 1-bromo-4-methylbenzene (0.8552 g, 5 mmol, 1 eq.) and benzylamine (0.822 mL, 7.5 mmol, 1.5 eq.) in 89 % yield.

^{1}H NMR (400 MHz, $CDCl_3$) δ 7.52 - 7.37 (m, 5H, Ar), 7.16 - 7.11 (dt, 2H, *J* = 8 Hz, Ar), 6.72 - 6.67 (dt, 2H, *J* = 8 Hz, Ar), 4.41 (s, 2H, CH_2), 3.97 (s, 1H, NH), 2.40 (s, 3H, CH_3).

This compound has been previously reported.[5]

3c

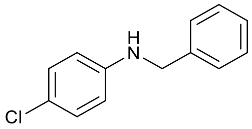

N-Benzyl-4-chloroaniline (**3c**) was formed from 1-bromo-4-chlorobenzene (0.9573 g, 5 mmol, 1 eq.) and benzylamine (0.822 mL, 7.5 mmol, 1.5 eq.) in 78 % yield.

^{1}H NMR (400 MHz, $CDCl_3$) δ 7.43 - 7.33 (m, 5H, Ar), 7.23 - 7.18 (m, 2H, Ar), 6.62 - 6.57 (m, 2H, Ar), 4.34 (s, 2H, CH_2), 3.93 (s, 1H, NH).

This compound has been previously reported.[5]

3d

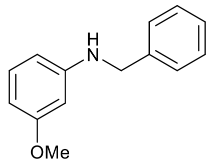

N-Benzyl-3-methoxyaniline (**3d**) was formed from 1-bromo-3-methoxybenzene (0.633 mL, 5 mmol, 1 eq.) and benzylamine (0.822 mL, 7.5 mmol, 1.5 eq.) in 89 % yield.

^{1}H NMR (400 MHz, $CDCl_3$) δ 7.46 - 7.35 (m, 5H, Ar), 7.21 - 7.16 (t, 1H, *J* = 16 Hz, *J* = 8 Hz, Ar), 6.42 - 6.38 (dd, *J* = 2 Hz, *J* = 8 Hz, 1H, Ar), 6.37 - 6.33 (dd, *J* = 2 Hz, *J* = 8 Hz, 1H, Ar), 6.31 - 6.28 (t, 1H, *J* = 2 Hz, *J* = 4 Hz, Ar), 4.38 (s, 2H, CH_2), 3.94 (s, 1H, NH), 3.83 (s, 3H, CH_3).

This compound has been previously reported.[5]

3e

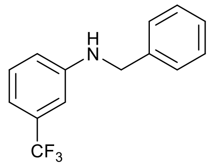

N-Benzyl-3-(trifluoromethyl)aniline (**3e**) was formed from 3-bromobenzotrifluoride (0.791 mL, 5 mmol, 1 eq.) and benzylamine (0.822 mL, 7.5 mmol, 1.5 eq.) in 92 % yield.

^{1}H NMR (400 MHz, $CDCl_3$) δ 7.42 - 7.26 (m, 5H, Ar), 7.05 - 7.00 (d, 1H, *J* = 8 Hz, Ar), 6.93 - 6.89 (m, 1H, Ar), 6.81 - 6.75 (m, 1H, Ar), 4.37 (s, 2H, CH_2), 3.92 (s, 1H, NH).

This compound has been previously reported.[5]

3f

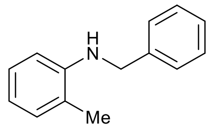

N-Benzyl-2-methylaniline (**3f**) was formed from 2-bromotoluene (0.604 mL, 5 mmol, 1 eq.) and benzylamine (0.822 mL, 7.5 mmol, 1.5 eq.) in 9 % yield.

^{1}H NMR (400 MHz, $CDCl_3$) δ 6.90 – 6.84 (m, 1H, Ar), 6.81 – 6.76 (m, 2H, Ar), 6.71 – 6.68 (d, 1H, *J* = 8 Hz, Ar), 4.43 (s, 2H, CH_2), 2.24 (s, 3H, CH_3).

This compound has been previously reported.[5]

3g

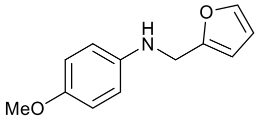

N-(Furan-2-ylmethyl)-4-methoxyaniline (**3g**) was formed from 4-bromoanisole (0.626 mL, 5 mmol, 1 eq.) and furfurylamine (0.663 mL, 7.5 mmol, 1.5 eq.) in 99 % yield.

^{1}H NMR (400 MHz, $CDCl_3$) δ 7.44 – 7.40 (m, 1H, Ar), 6.88 – 6.83 (dt, 2H, *J* = 8 Hz, Ar), 6.73 – 6.66 (dt, 2H, *J* = 8 Hz, Ar), 6.40 – 6.36 (m, 1H, Ar), 6.30 – 6.25 (m, 1H, Ar), 4.32 (s, 2H, CH_2), 3.87 (s, 1H, NH), 3.80 (s, 3H, CH_3).

This compound has been previously reported.[6]

3h

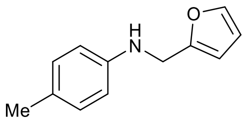

N-(Furan-2-ylmethyl)-4-methylaniline (**3h**) was formed from 4-bromotoluene (0.8552 g, 5 mmol, 1 eq.) and furfurylamine (0.663 mL, 7.5 mmol, 1.5 eq.) in 14 % yield.

^{1}H NMR (400 MHz, $CDCl_3$) δ 7.03 – 7.02 (m, 1H, Ar), 6.65 – 6.61 (dt, 2H, *J* = 8 Hz, Ar), 6.26 – 6.24 (m, 1H, Ar), 4.31 (s, 2H, CH_2), 2.28 (s, 3H, CH_3).

This compound has been previously reported.[1]

3i

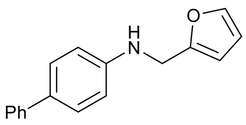

N-(Furan-2-ylmethyl)-[1,1'-biphenyl]-4-amine (**3i**) was formed from 4-bromobiphenyl (1.1655 g, 5 mmol, 1 eq.) and furfurylamine (0.663 mL, 7.5 mmol, 1.5 eq.) in 71 % yield.

^{1}H NMR (400 MHz, $CDCl_3$) δ 7.60 – 7.57 (m, 2H, Ar), 7.49 – 7.39 (m, 5H, Ar), 7.34 – 7.27 (m, 1H, Ar), 6.82 – 6.76 (dt, 2H, *J* = 8 Hz, Ar), 6.40 – 6.37 (m, 1H, Ar), 6.33 – 6.29 (m, 1H, Ar), 4.40 (s, 2H, CH_2).

This compound has been previously reported.[1]

3j

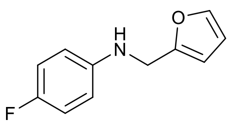

4-Fluoro-N-(furan-2-ylmethyl)aniline (**3j**) was formed from 1-bromo-4-fluorobenzene (0.549 mL, 5 mmol, 1 eq.) and furfurylamine (0.663 mL, 7.5 mmol, 1.5 eq.) in 12 % yield.

^{1}H NMR (400 MHz, $CDCl_3$) δ 7.39 – 7.38 (m, 1H, Ar), 6.92 – 6.87 (dt, 2H, *J* = 8 Hz, Ar), 6.80 – 6.76 (dt, 2H, *J* = 8 Hz, Ar), 6.63 – 6.58 (m, 2H, Ar), 4.27 (s, 2H, CH_2).

This compound has been previously reported.[1]

3k

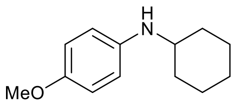

N-Cylcohexyl-4-methoxyaniline (**3k**) was formed from 4-bromoanisole (0.626 mL, 5 mmol, 1 eq.) and cyclohexylamine (0.858 mL, 7.5 mmol, 1.5 eq.) in 14 % yield.

^{1}H NMR (400 MHz, $CDCl_3$) δ 6.62 – 6.55 (dt, 2H, *J* = 8 Hz, Ar), 3.74 (s, 3H, CH_3), 3.23 – 3.12 (m, 1H, CH).

This compound has been previously reported.[1]

3l

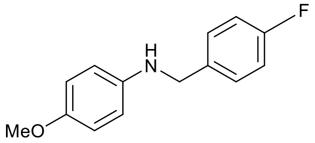

N-(4-Fluorobenzyl)-4-methoxyaniline (**3l**) was formed from 4-bromoanisole (0.626 mL, 5 mmol, 1 eq.) and 4-fluorobenzylamine (0.857 mL, 7.5 mmol, 1.5 eq.) in 99 % yield.

^{1}H NMR (400 MHz, $CDCl_3$) δ 7.41 – 7.35 (m, 2H, Ar), 7.12 – 7.04 (tt, 3H, *J* = 12 Hz, *J* = 3.2 Hz, Ar), 6.88 – 6.83 (dt, 2H, *J* = 4 Hz, Ar), 6.67 – 6.61 (dt, 2H, *J* = 4 Hz, Ar), 4.28 (s, 2H, CH_2), 3.88 (s, 1H, NH), 3.80 (s, 3H, CH_3).

This compound has been previously reported.[1]

3m

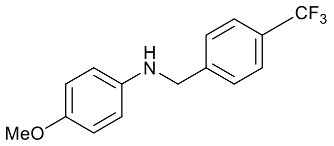

4-Methoxy-N-(4-(trifluoromethyl)benzyl)aniline (**3m**) was formed from 4-bromoanisole (0.626 mL, 5 mmol, 1 eq.) and 4-trifluorobenzylamine (1.070 mL, 7.5 mmol, 1.5 eq.) in 41 % yield.

^{1}H NMR (400 MHz, $CDCl_3$) δ 7.35 – 7.30 (d, 2H, *J* = 8 Hz, Ar), 7.30 – 7.24 (dt, 2H, *J* = 12 Hz, Ar), 6.71 – 6.65 (m, 2H, Ar), 6.50 – 6.43 (dt, 2H, *J* = 8 Hz, Ar), 4.25 (s, 2H, CH_2), 3.63 (s, 3H, CH_3).

This compound has been previously reported.[7]

3n

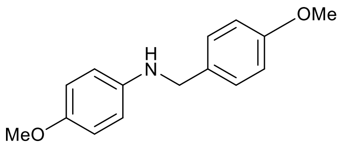

4-Methoxy-N-(4-methxybenzyl)aniline (**3n**) was formed from 4-bromoanisole (0.626 mL, 5 mmol, 1 eq.) and 4-methoxybenzylamine (0.980 mL, 7.5 mmol, 1.5 eq.) in 22 % yield.

^{1}H NMR (400 MHz, $CDCl_3$) δ 8.18 (s, 1H, NH), 7.63 – 7.59 (dt, 2H, *J* = 8 Hz, Ar), 6.84 – 6.80 (m, 2H, Ar), 4.62 (s, 2H, CH_2), 3.71 (s, 6H, CH_3).

This compound has been previously reported.[8]

3p

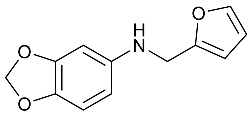

N-(Furan-2-ylmethyl)-4-methylaniline (**3o**) was formed from 1-bromo-3,4-(methylenedioxy)benzene (0.602 mL, 5 mmol, 1 eq.) and furfurylamine (0.663 mL, 7.5 mmol, 1.5 eq.) in 13 % yield.

^{1}H NMR (400 MHz, $CDCl_3$) δ 6.56 – 6.53 (s, 1H, Ar), 6.12 – 6.10 (m, 1H, Ar), 6.02 – 5.98 (m, 2H, Ar), 5.74 (s, 2H, CH_2), 4.13 (s, 2H, CH_2).

This compound has been previously reported.[1]

3q

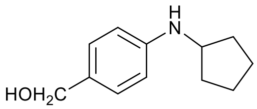

(4-(Cylcopentylamino)phenyl)methanol (**3p**) was formed from 4-bromobenzylalcohol (0.9352 g, 5 mmol, 1 eq.) and cyclopentylamine (0.740 mL, 7.5 mmol, 1.5 eq.) in 30 % yield.

^{1}H NMR (400 MHz, $CDCl_3$) δ 7.10 – 7.04 (d, 2H, *J* = 8 Hz, Ar), 6.53 – 6.46 (d, 2H, *J* = 8 Hz, Ar), 4.44 (s, 2H, CH_2), 3.74 (m, 1H, CH), 1.99 – 1.86 (m, 2H, CH_2), 1.41 – 1.27 (m, 2H, CH_2).

This compound has been previously reported.[1]

3r

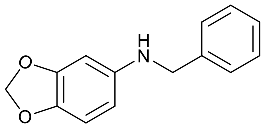

N-Benzylbenzo[d][1,3]dioxol-5-amine (**3q**) was formed from 1-bromo-3,4-(methylenedioxy)benzene (0.602 mL, 5 mmol, 1 eq.) and benzylamine (0.822 mL, 7.5 mmol, 1.5 eq.) in 68 % yield.

^{1}H NMR (400 MHz, $CDCl_3$) δ 7.25 – 7.10 (m, 5H, Ar), 6.14 – 6.11 (d, 1H, *J* = 4 Hz, Ar), 5.95 – 5.89 (dd, 1H, *J* = 2 Hz, *J* = 8 Hz, Ar), 5.78 (s, 1H, Ar), 5.68 (s, 2H, CH_2), 4.10 (s, 2H, CH_2).

This compound has been previously reported.[9]

3s

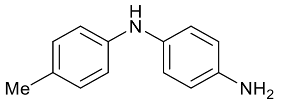

N[1]-(*p*-Tolyl)-benzene-1,4-diamine (**3r**) was formed from 1-bromo-4-methylbenzene (0.8552 mL, 5 mmol, 1 eq.) and *p*-phenylenediamine (0.8111 g, 7.5 mmol, 1.5 eq.) in 23 % yield.

^{1}H NMR (400 MHz, $CDCl_3$) δ 7.06 – 7.01 (m, 2H, Ar), 6.99 – 6.95 (dt, 2H, *J* = 8 Hz, Ar), 6.84 – 6.81 (dt, 2H, *J* = 8 Hz, Ar), 6.71 – 6.66 (dt, 2H, *J* = 8 Hz, Ar), 5.34 (s, 1H, NH), 2.33 (s, 3H, CH_3).

This compound has been previously reported.[1]

3t

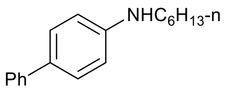

N-Hexyl-[1,1'-biphenyl]-4-amine (**3s**) was formed from 4-bromobiphenyl (1.1655 g, 5 mmol, 1 eq.) and hexylamine (0.991 mL, 7.5 mmol, 1.5 eq.) in 75 % yield.

^{1}H NMR (400 MHz, $CDCl_3$) δ 7.35 – 7.27 (m, 1H, Ar), 6.77 – 6.69 (dt, 2H, *J* = 12 Hz, Ar), 3.24 – 3.15 (t, 2H, *J* = 7 Hz, *J* = 14 Hz, CH_2), 2.78 – 2.69 (t, 2H, *J* = 8 Hz, *J* = 12 Hz, CH_2), 1.73 – 1.64 (m, 2H, CH_2), 1.54 – 1.45 (m, 4H, CH).

This compound has been previously reported.[1]

3u

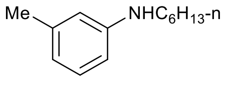

N-Hexyl-3-methylaniline (**3t**) was formed from 3-bromotoluene (0.606 mL, 5 mmol, 1 eq.) and hexylamine (0.991 mL, 7.5 mmol, 1.5 eq.) in 51 % yield.

^{1}H NMR (400 MHz, $CDCl_3$) δ 7.36 (s, 1H, NH), 7.33 – 7.29 (m, 1H, Ar), 6.57 – 6.53 (d, 1H, *J* = 8 Hz, Ar), 6.47 – 6.42 (m, 2H, Ar), 3.14 – 3.08 (t, 2H, *J* = 4 Hz, *J* = 12 Hz, CH_2), 2.31 (s, 3H, CH_3), 1.67 – 1.57 (m, 2H, CH_2), 1.50 – 1.39 (m, 6H, CH).

This compound has been previously reported.[1]

3 NMR Spectra

3.1 ^{1}H and ^{13}C Spectra of Ligands

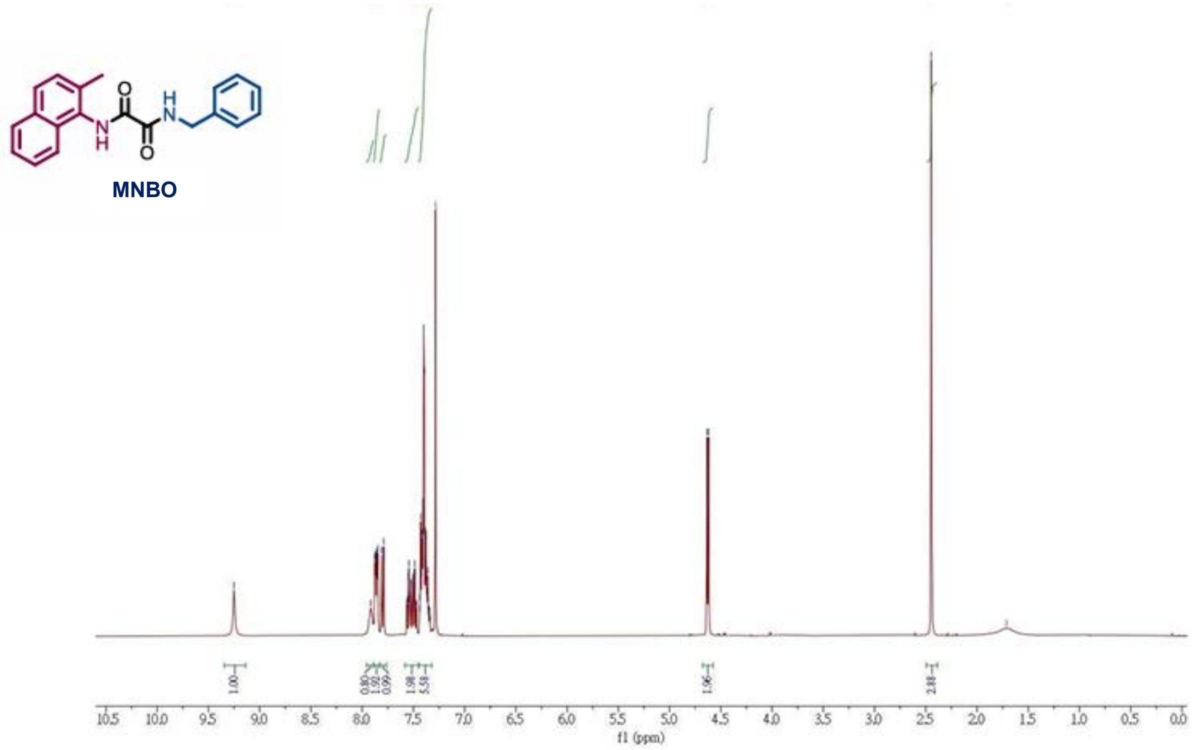

MNBO

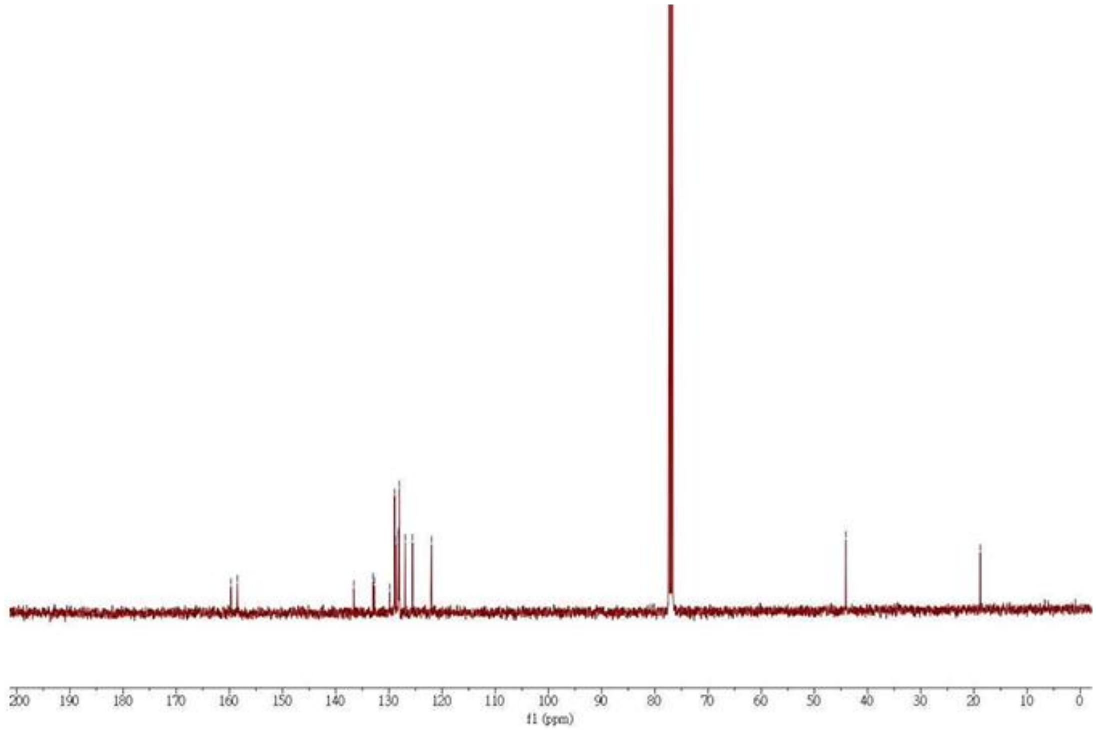

*Figure S15 NMR spectra of **MNBO**. Top ^{1}H (400 MHz, $CDCl_3$); bottom ^{13}C (100 MHz, $CDCl_3$)*

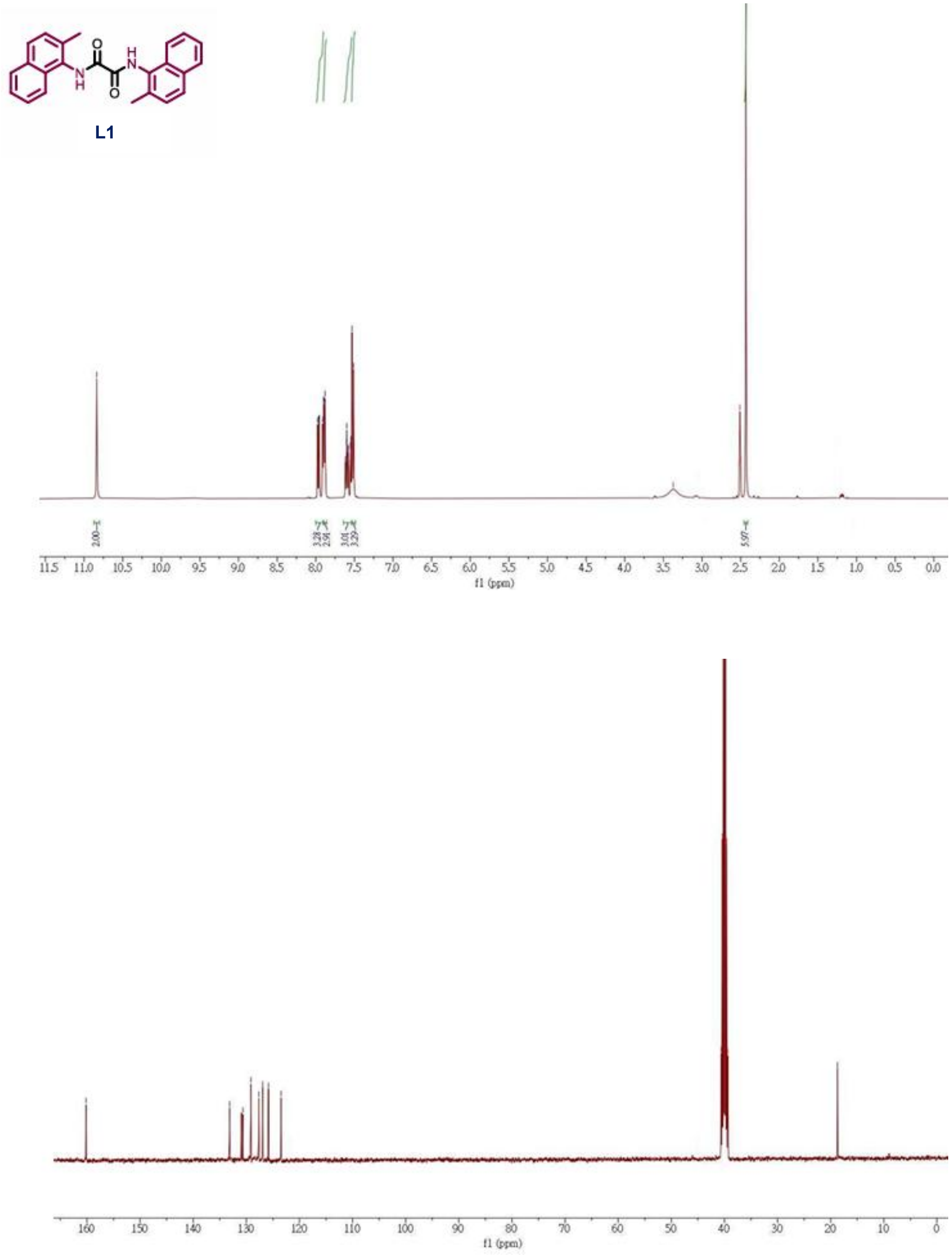

*Figure S16 NMR spectra of **L1**. Top ^{1}H (400 MHz, d6-DMSO); bottom ^{13}C (100 MHz, d6-DMSO)*

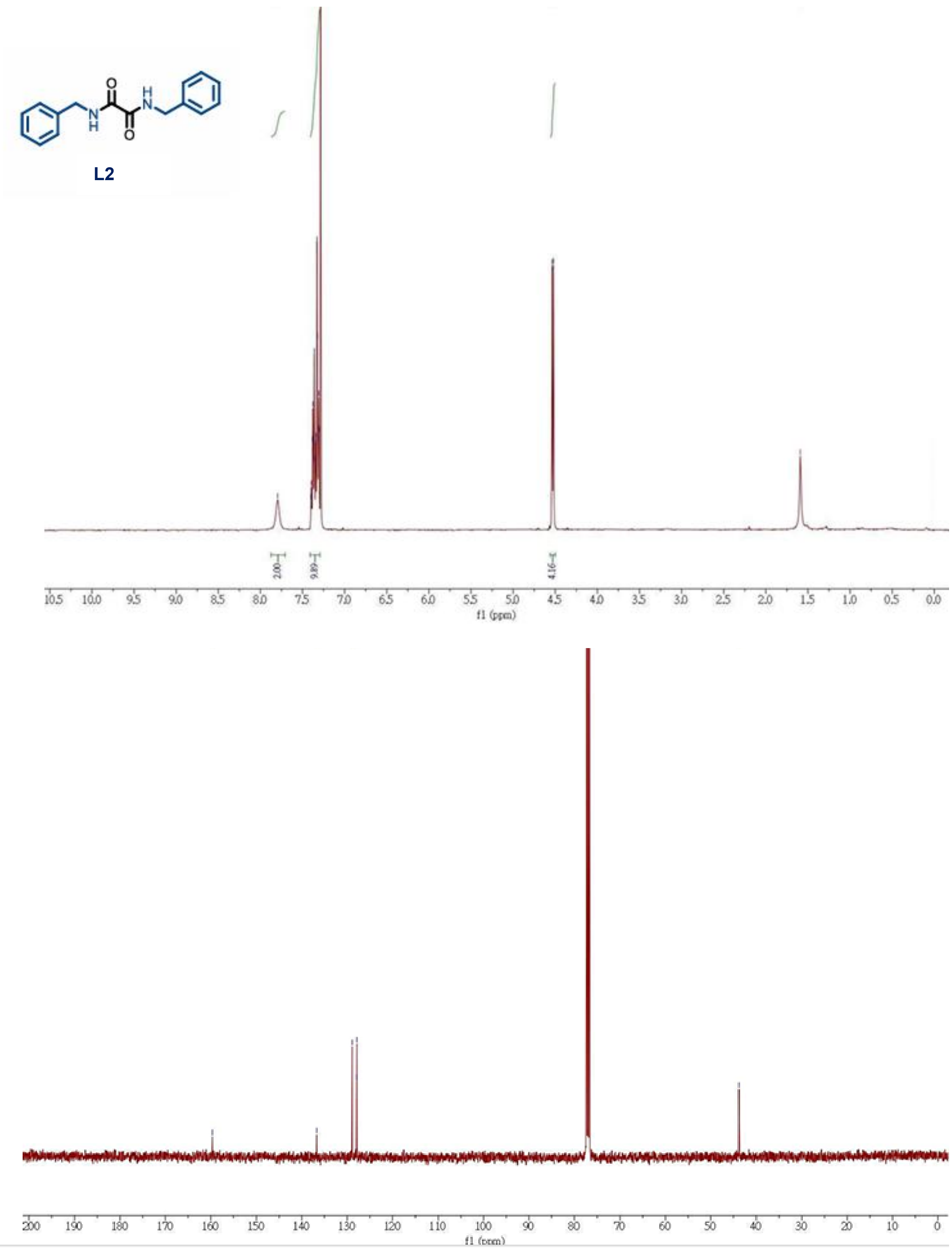

Figure S17 NMR spectra of ***L2****. Top* ^{1}*H (400 MHz,* $CDCl_3$*); bottom* ^{13}*C (100 MHz,* $CDCl_3$*)*

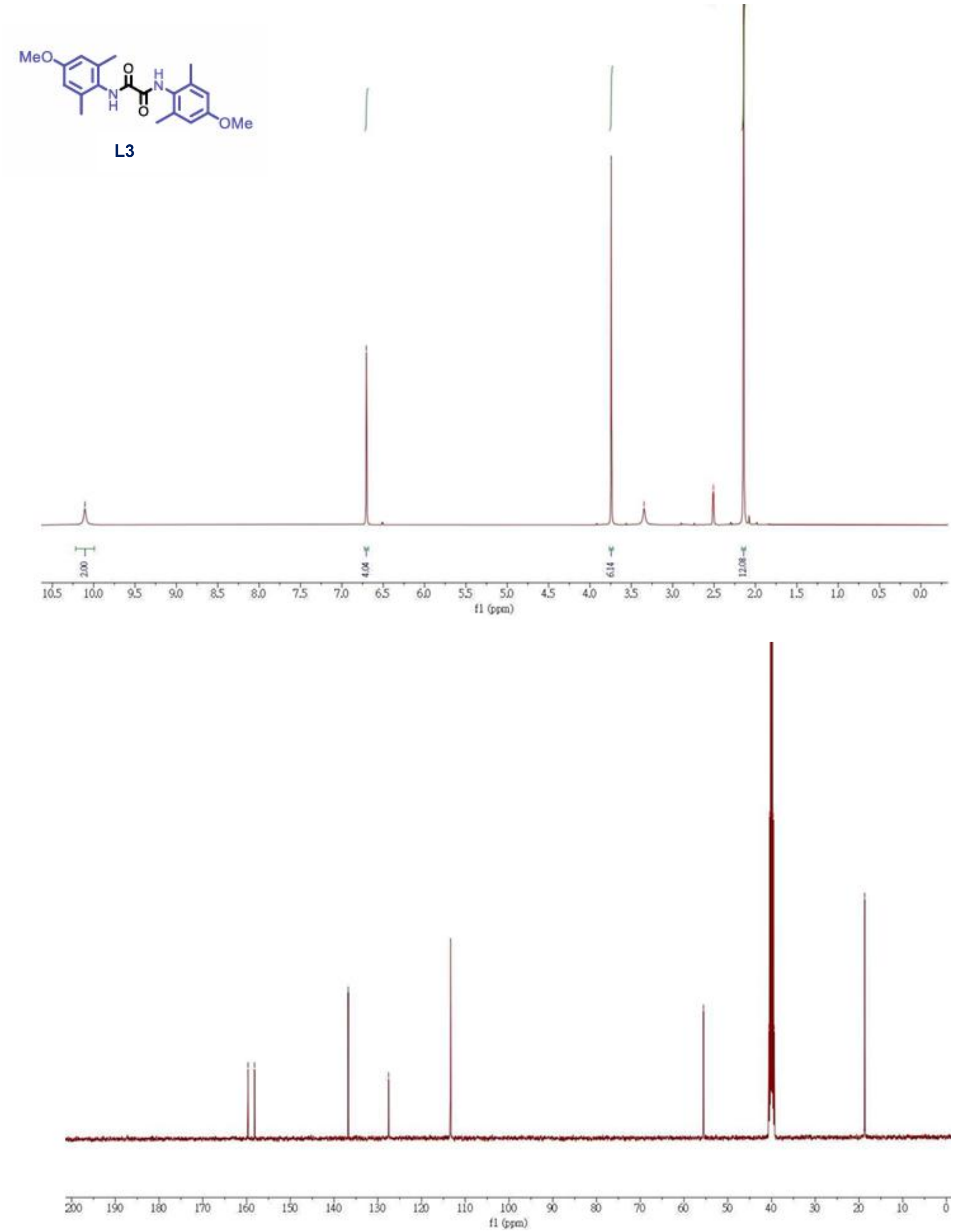

*Figure S18 NMR spectra of **L3**. Top ^{1}H (400 MHz, d6-DMSO); bottom ^{13}C (100 MHz, d6-DMSO).*

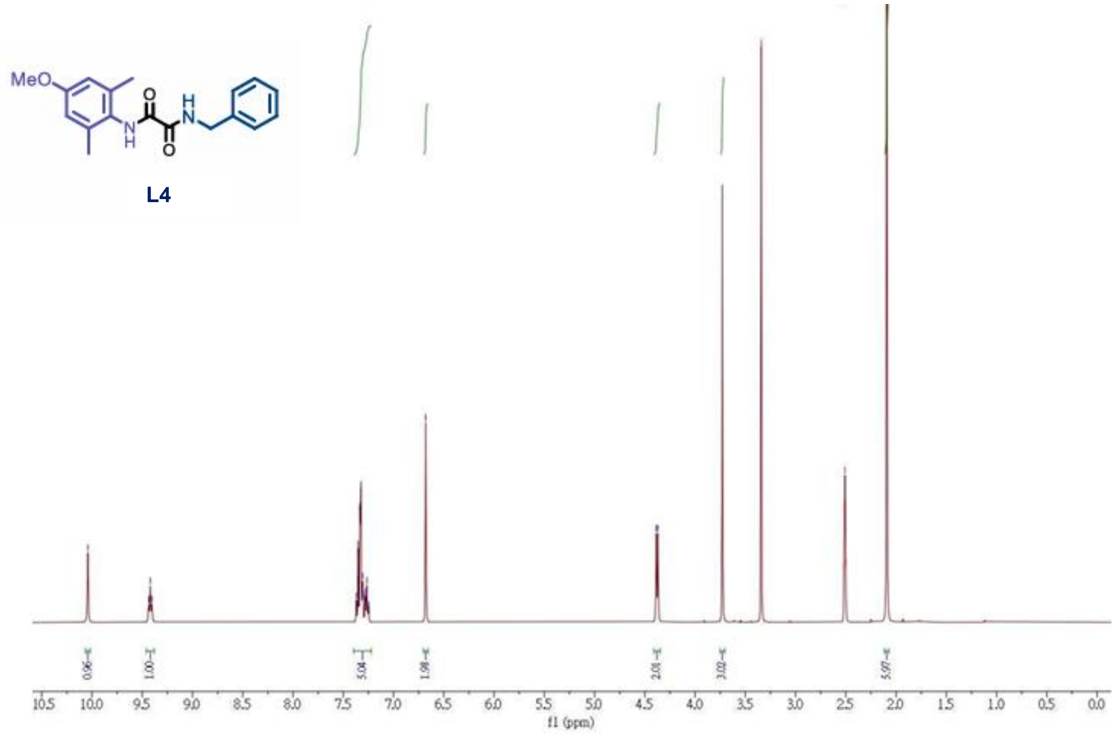

L4

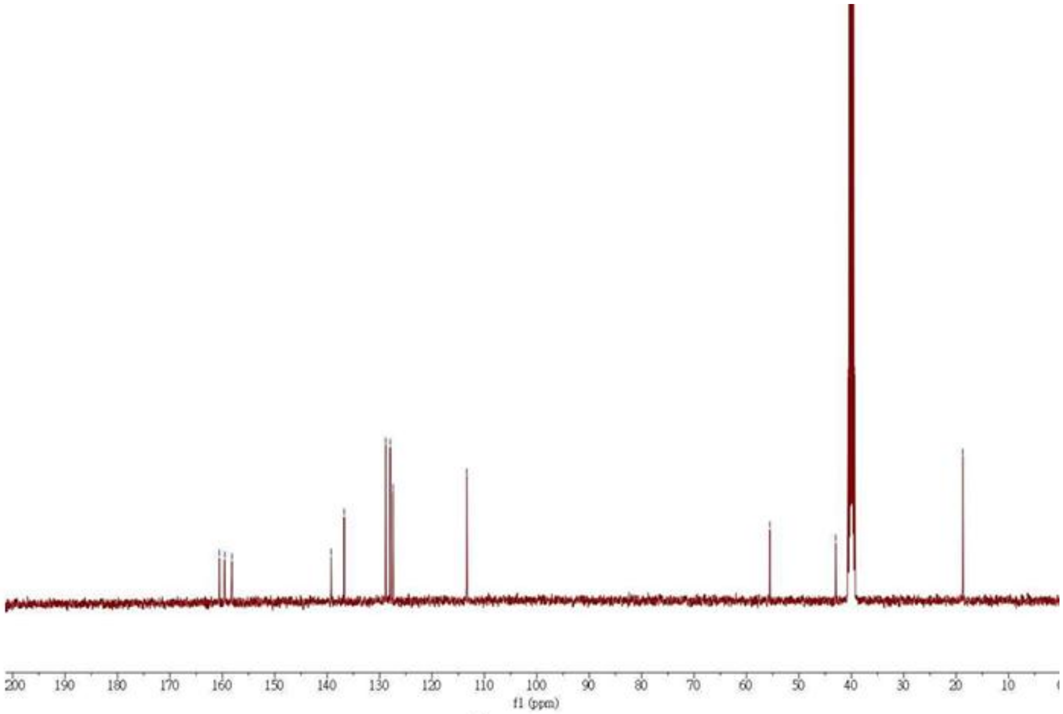

Figure S14: ^{13}C NMR spectrum of **L6**

*Figure S19 NMR spectra of **L4**. Top ^{1}H (400 MHz, d6-DMSO); bottom ^{13}C (100 MHz, d6-DMSO).*

3.2 [1]H Spectra for Reaction Scope Products

All NMR spectra recorded in $CDCl_3$ (400 MHz).

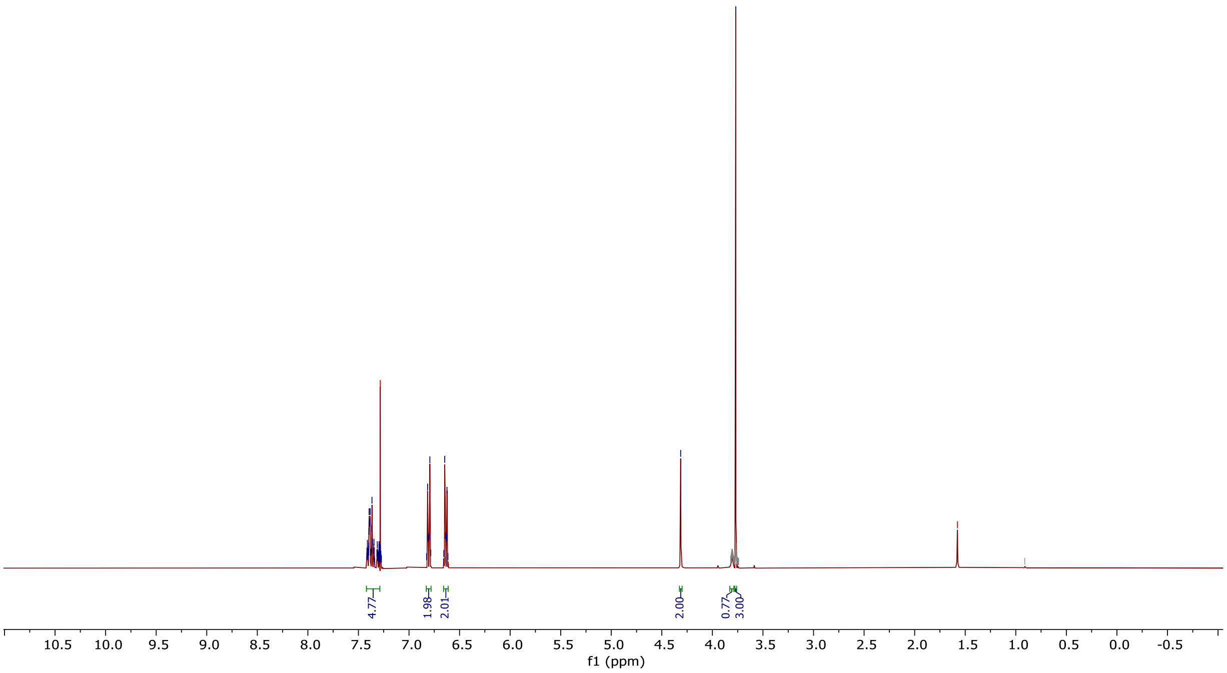

Figure S20 ^{1}H NMR of **3a** (isolated product from larger scale reaction).

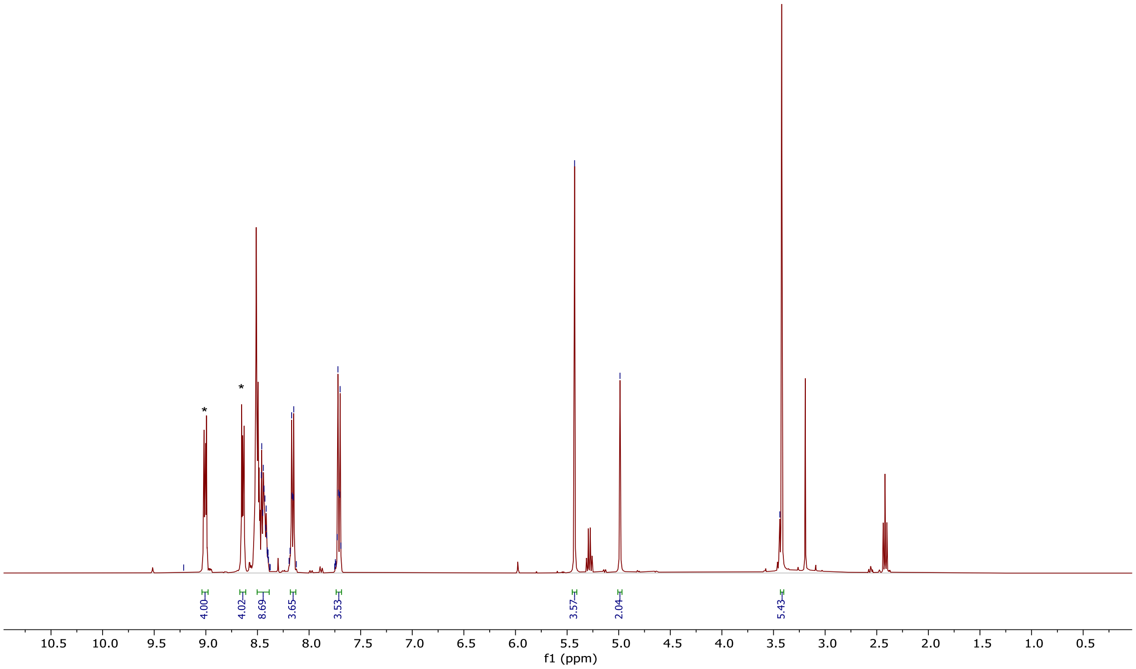

Figure S21 ^{1}H NMR of **3b** (crude product with naphthalene standard peaks labelled with *).

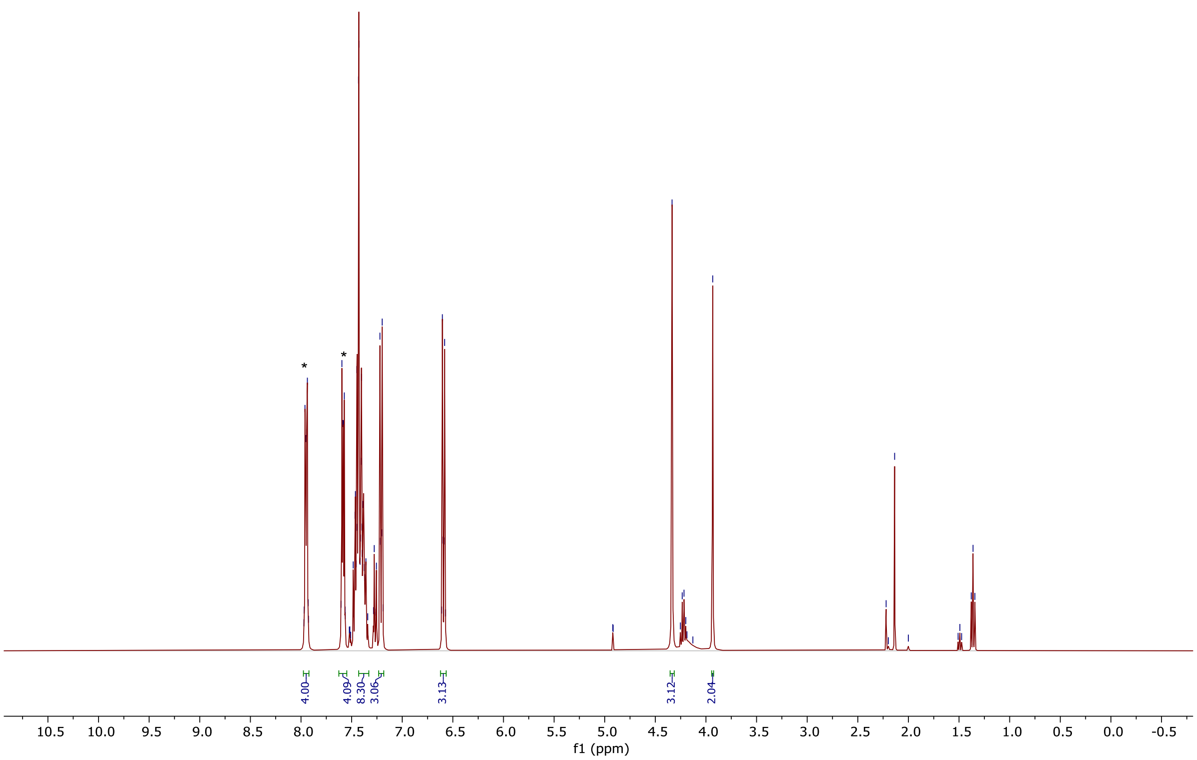

Figure S22 ^{1}H NMR of **3c** (crude product with naphthalene standard peaks labelled with *).

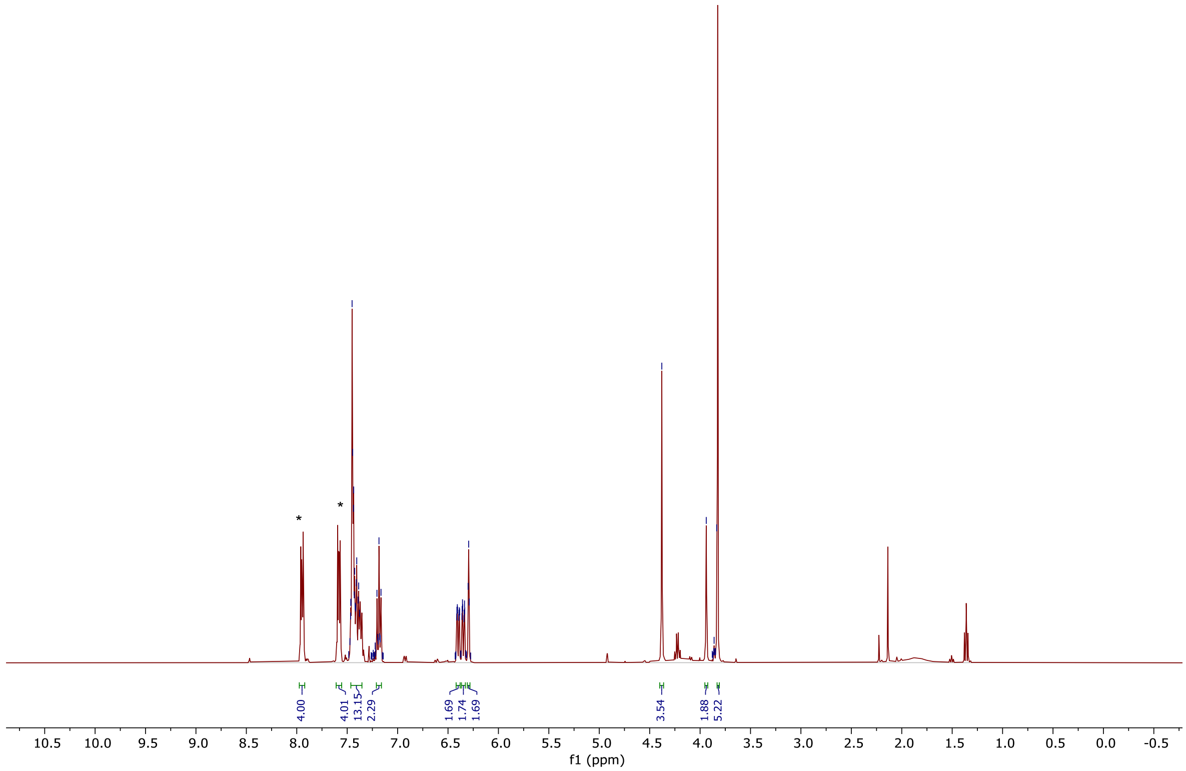

Figure S23 ^{1}H NMR of **3d** (crude product with naphthalene standard peaks labelled with *).

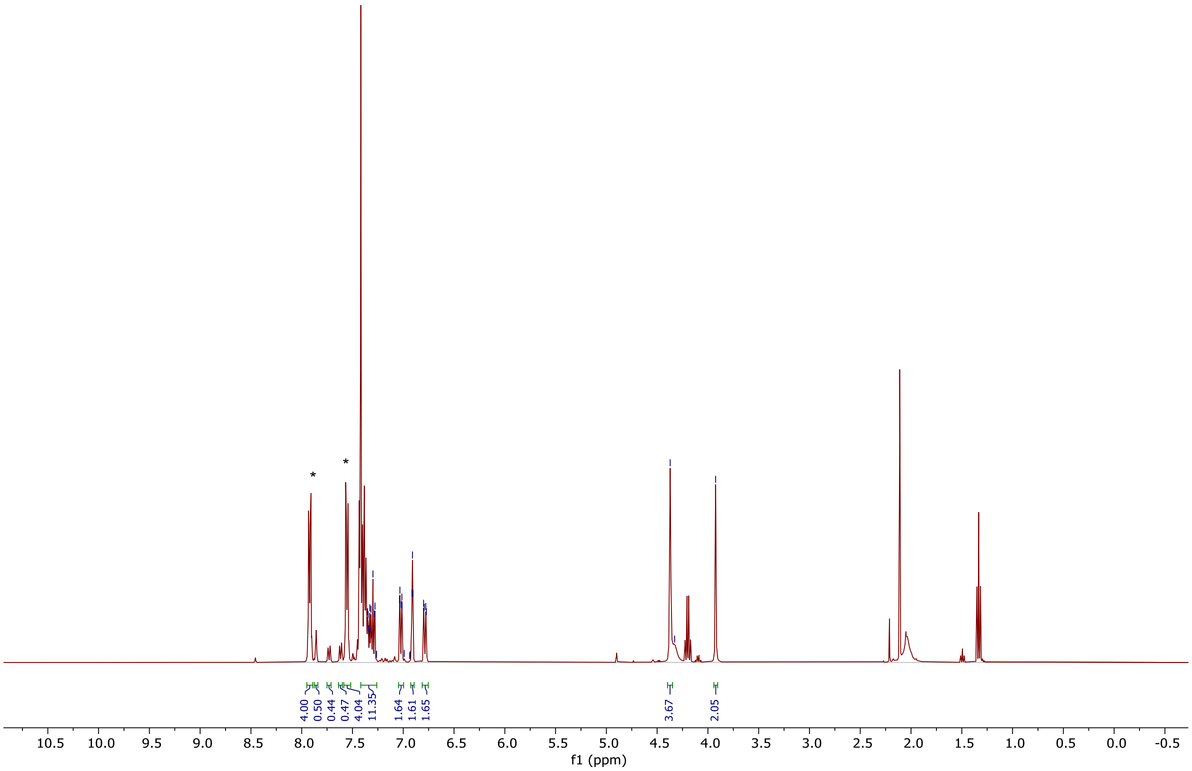

Figure S24 ¹H NMR of **3e** (crude product with naphthalene standard peaks labelled with *).

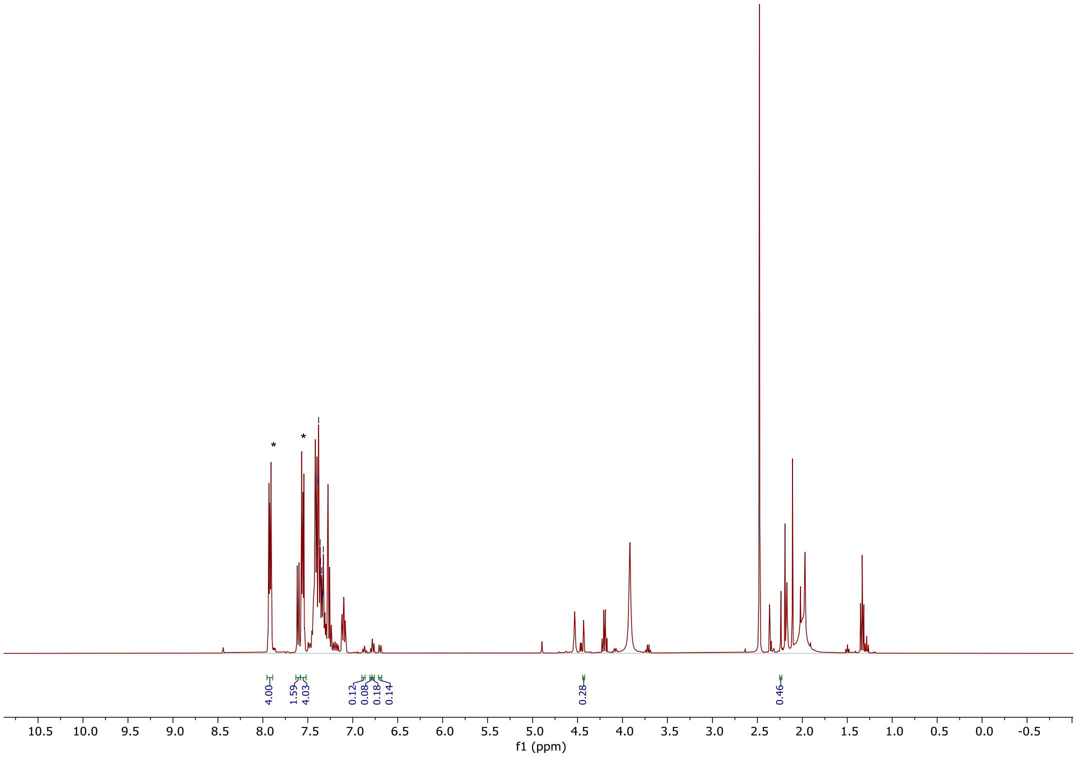

Figure S25 ¹H NMR of **3f** (crude product with naphthalene standard peaks labelled with *).

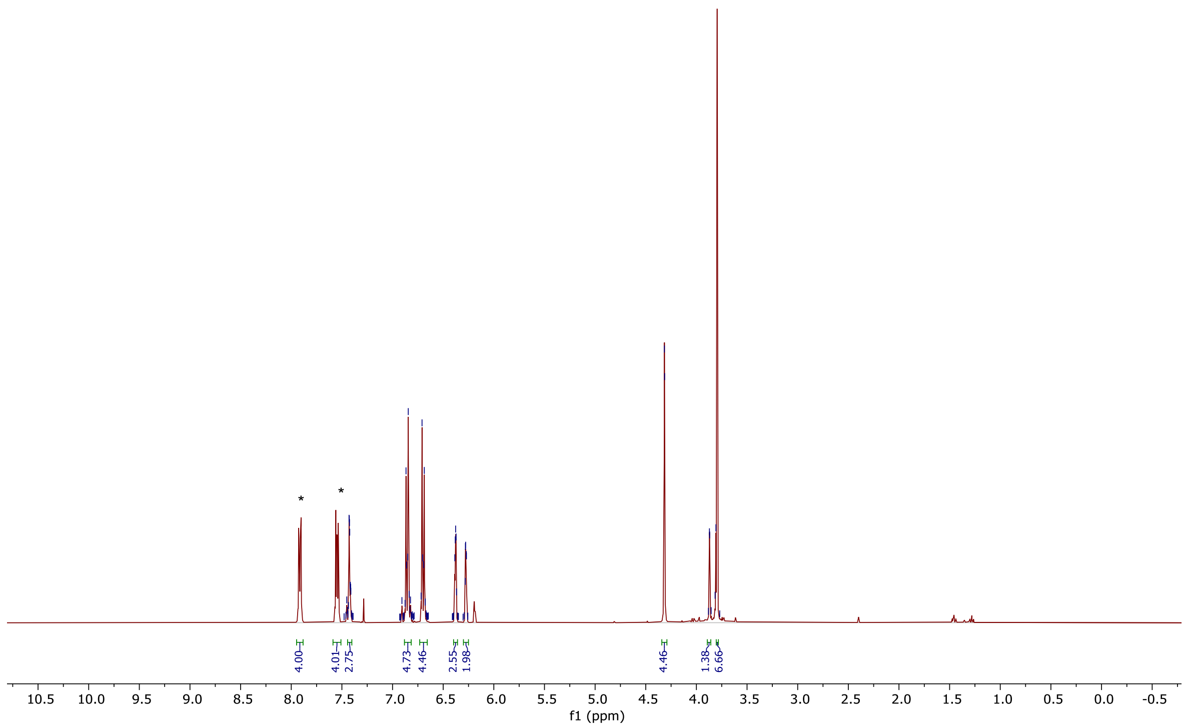

Figure S26 ^{1}H NMR of **3g** (crude product with naphthalene standard peaks labelled with *).

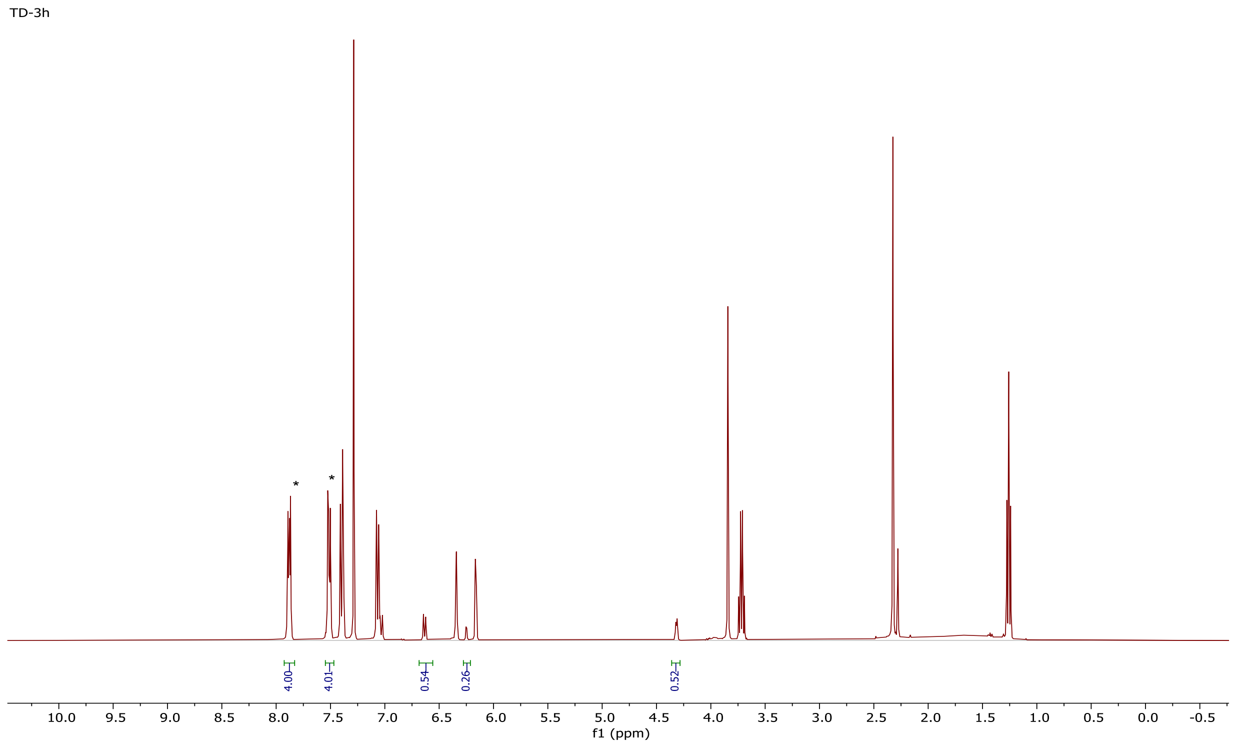

Figure S27 ^{1}H NMR of **3h** (crude product with naphthalene standard peaks labelled with *).

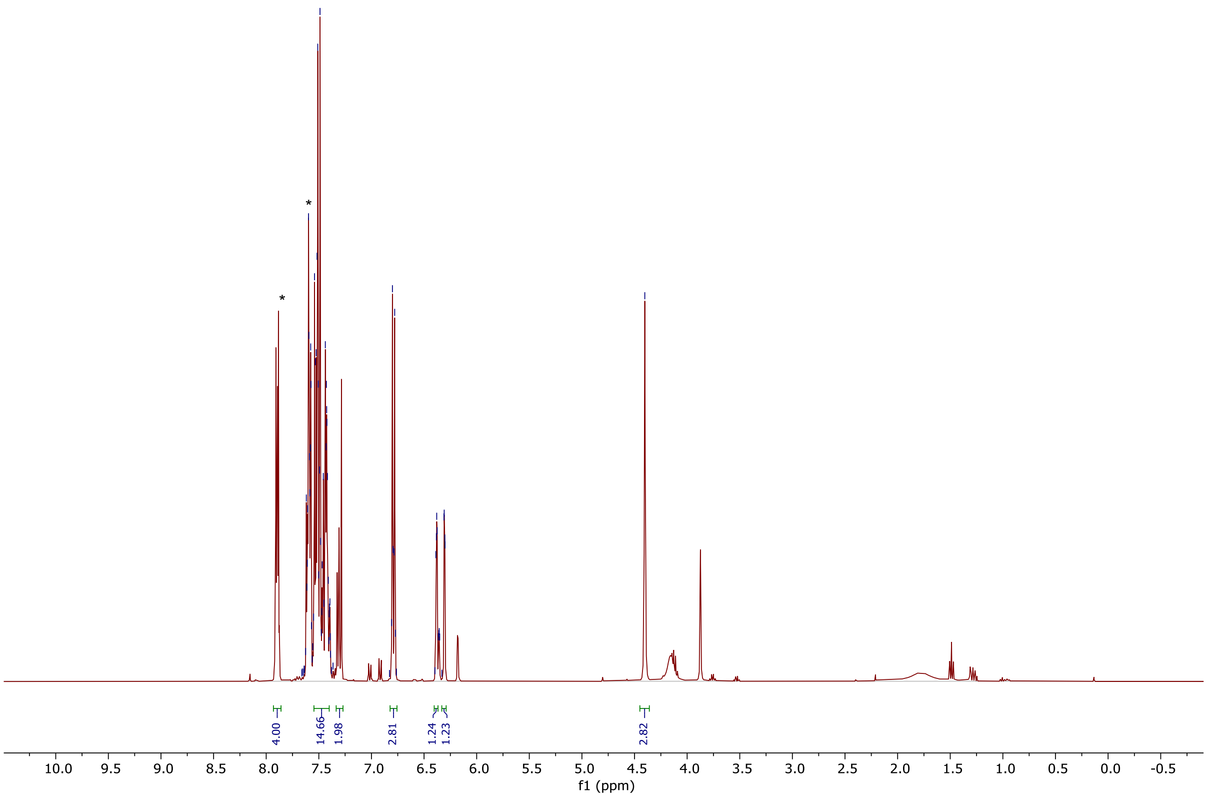

Figure S28 ^{1}H NMR of **3i** (crude product with naphthalene standard peaks labelled with *).

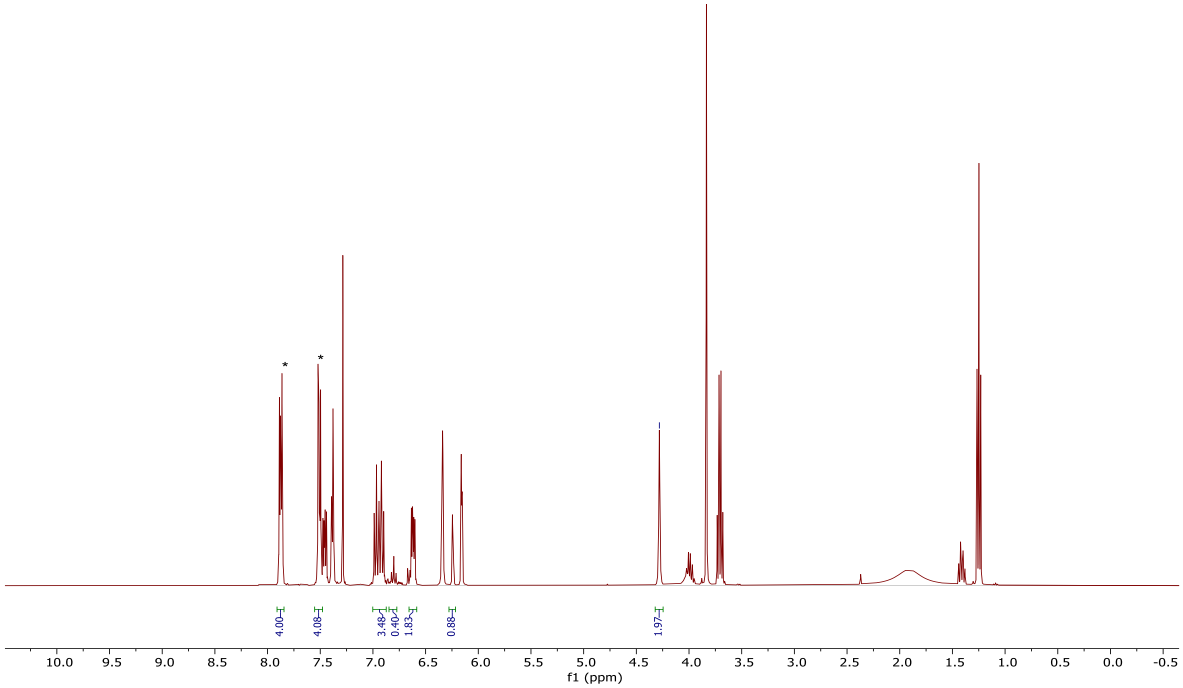

Figure S29 ^{1}H NMR of **3j** (crude product with naphthalene standard peaks labelled with *).

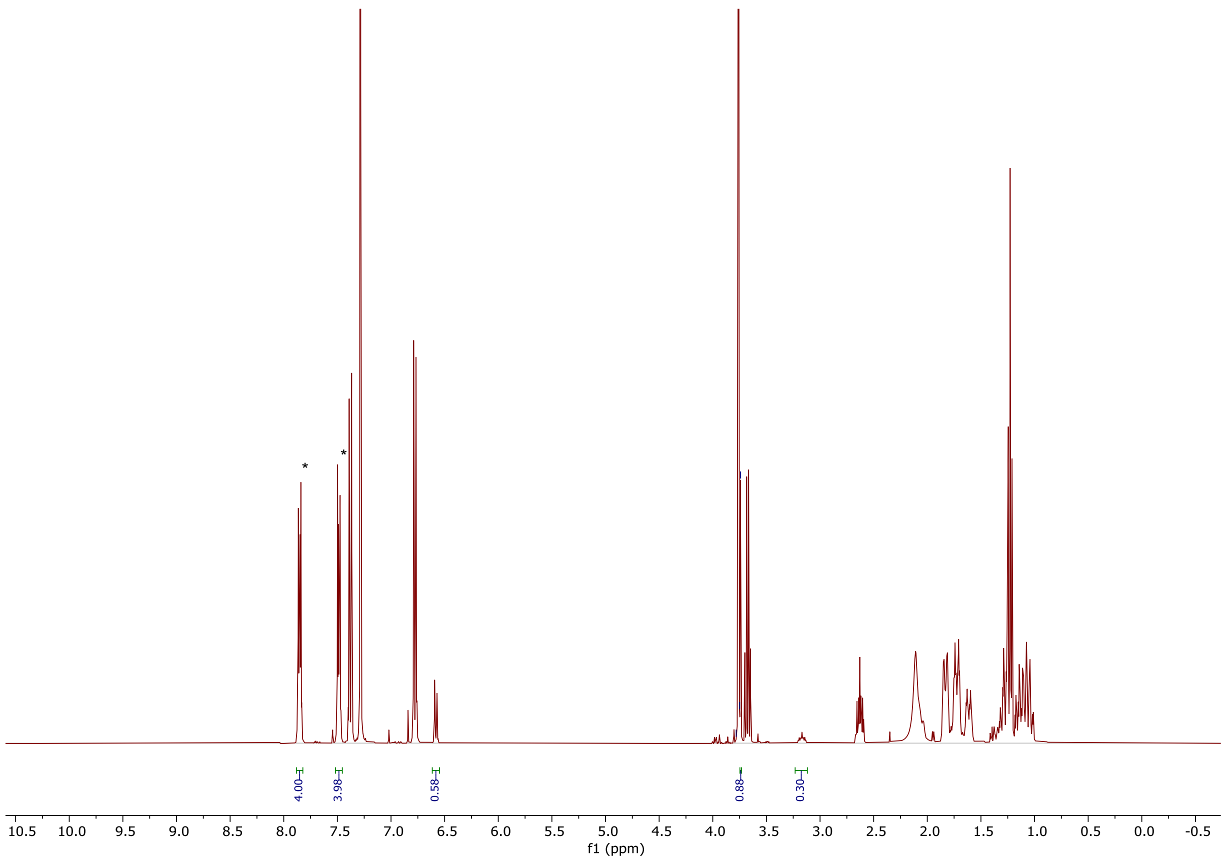

Figure S30 ^{1}H NMR of **3k** (crude product with naphthalene standard peaks labelled with *).

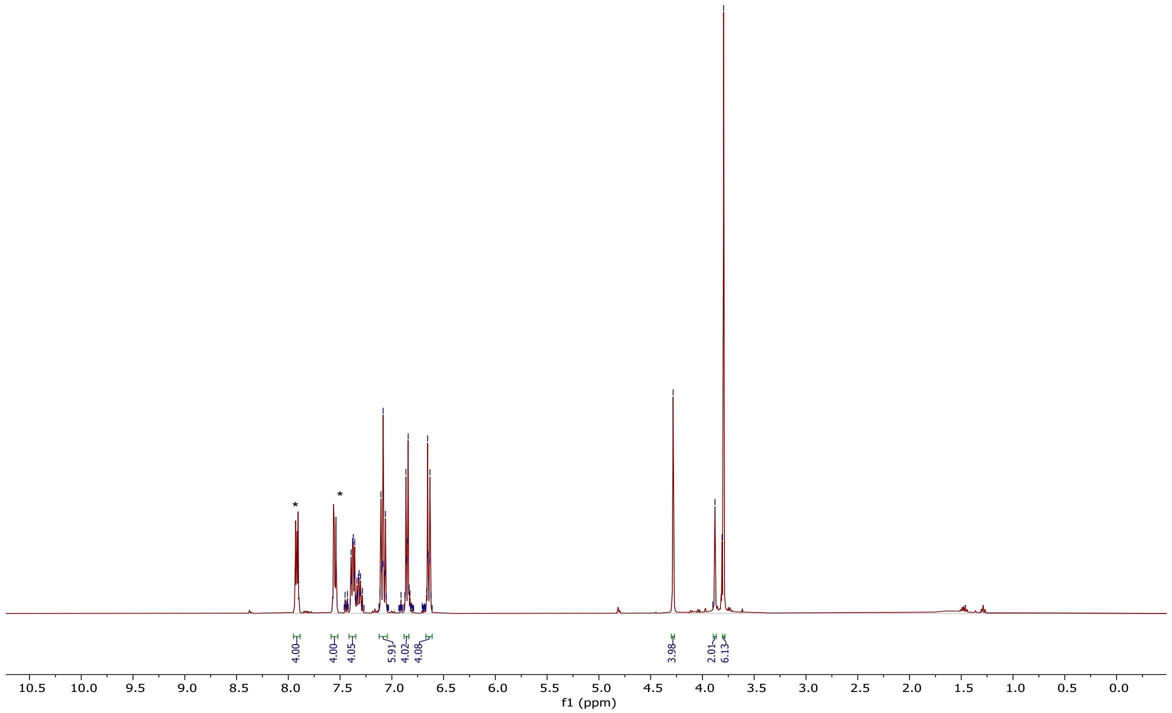

Figure S31 ^{1}H NMR of **3l** (crude product with naphthalene standard peaks labelled with *).

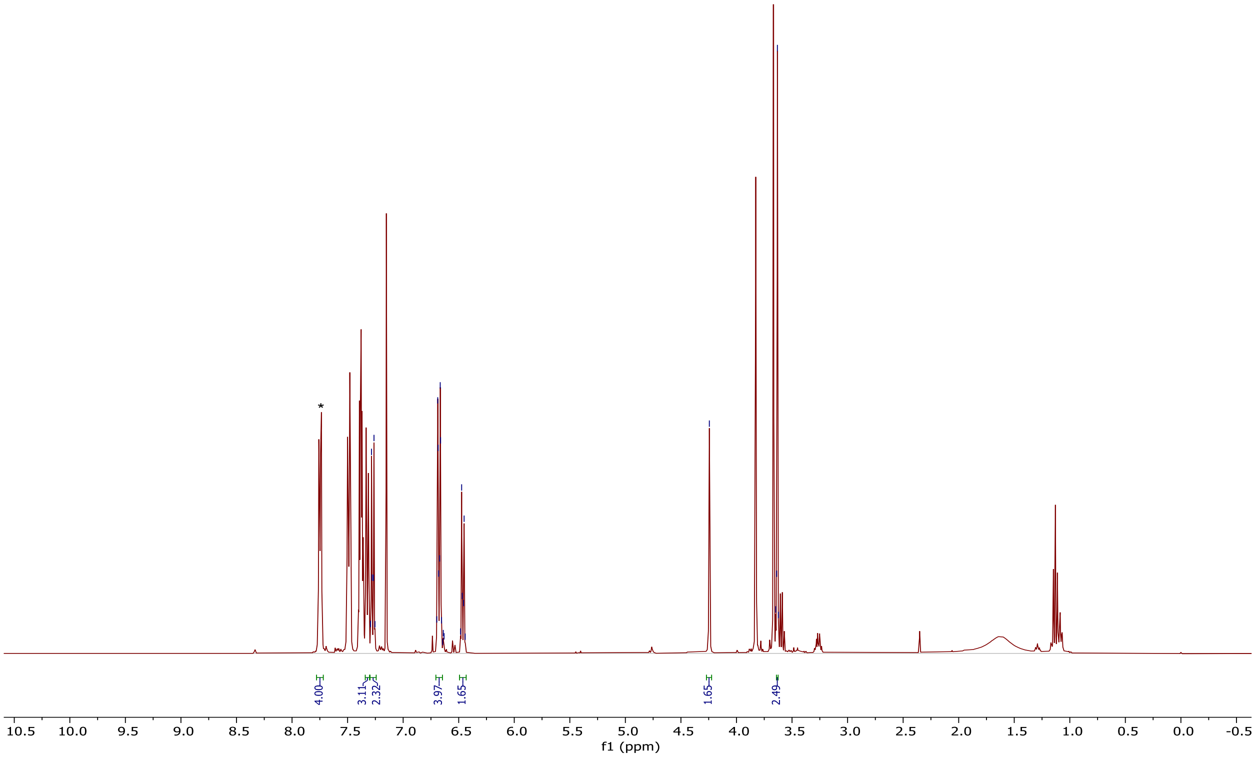

Figure S32 ^{1}H NMR of **3m** (crude product with naphthalene standard peaks labelled with *).

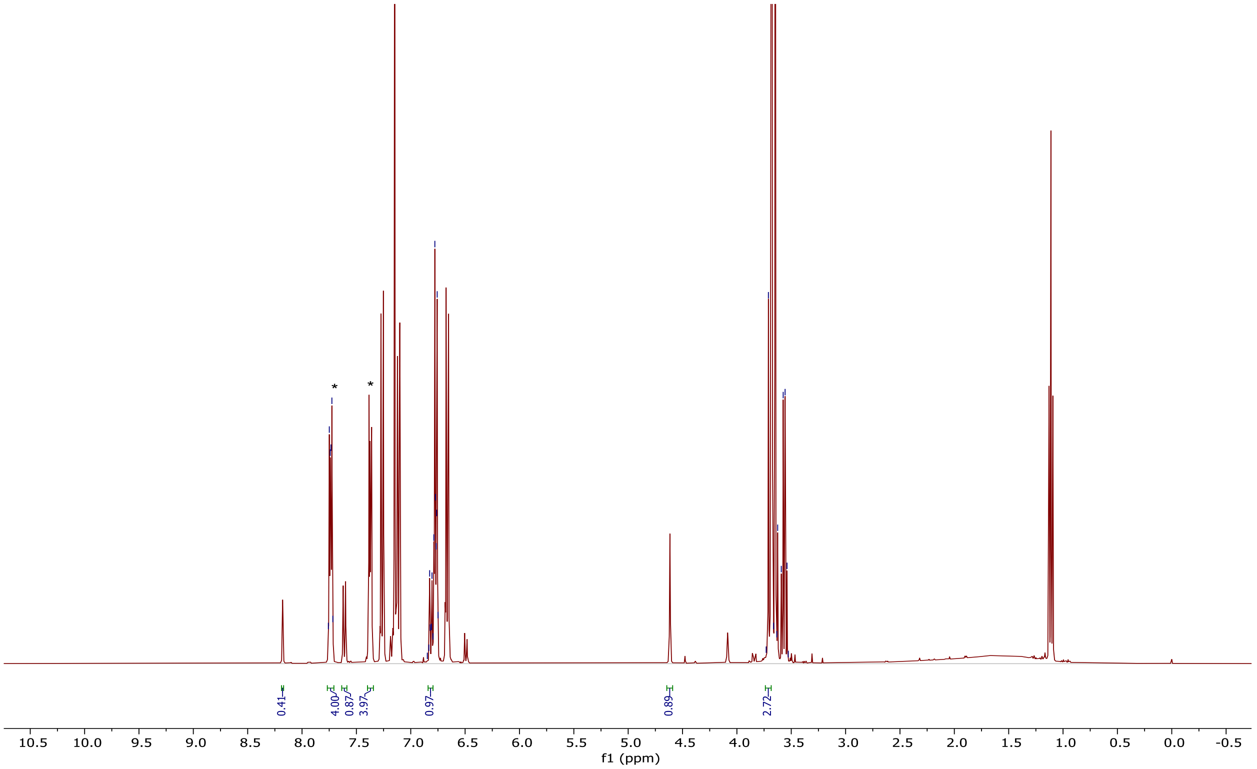

Figure S33 ^{1}H NMR of **3n** (crude product with naphthalene standard peaks labelled with *).

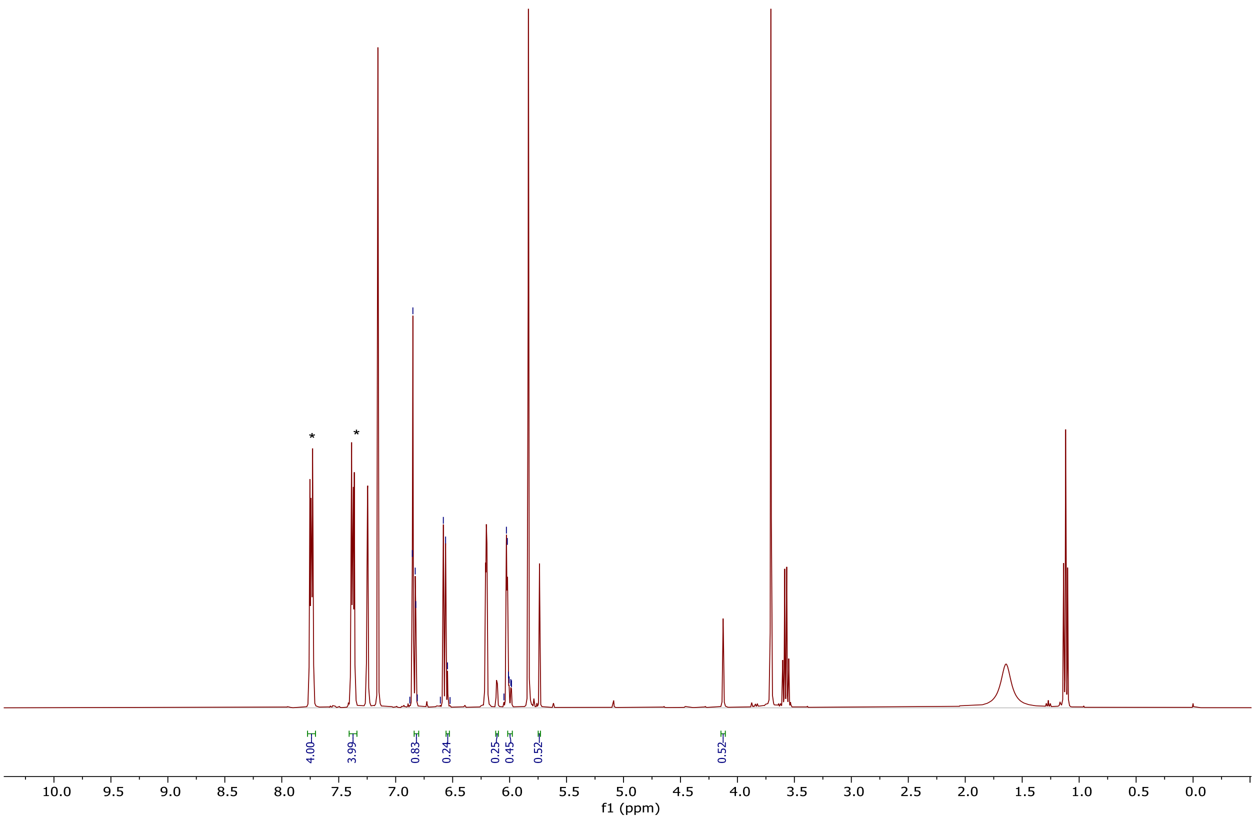

Figure S34 ^{1}H NMR of **3p** (crude product with naphthalene standard peaks labelled with *).

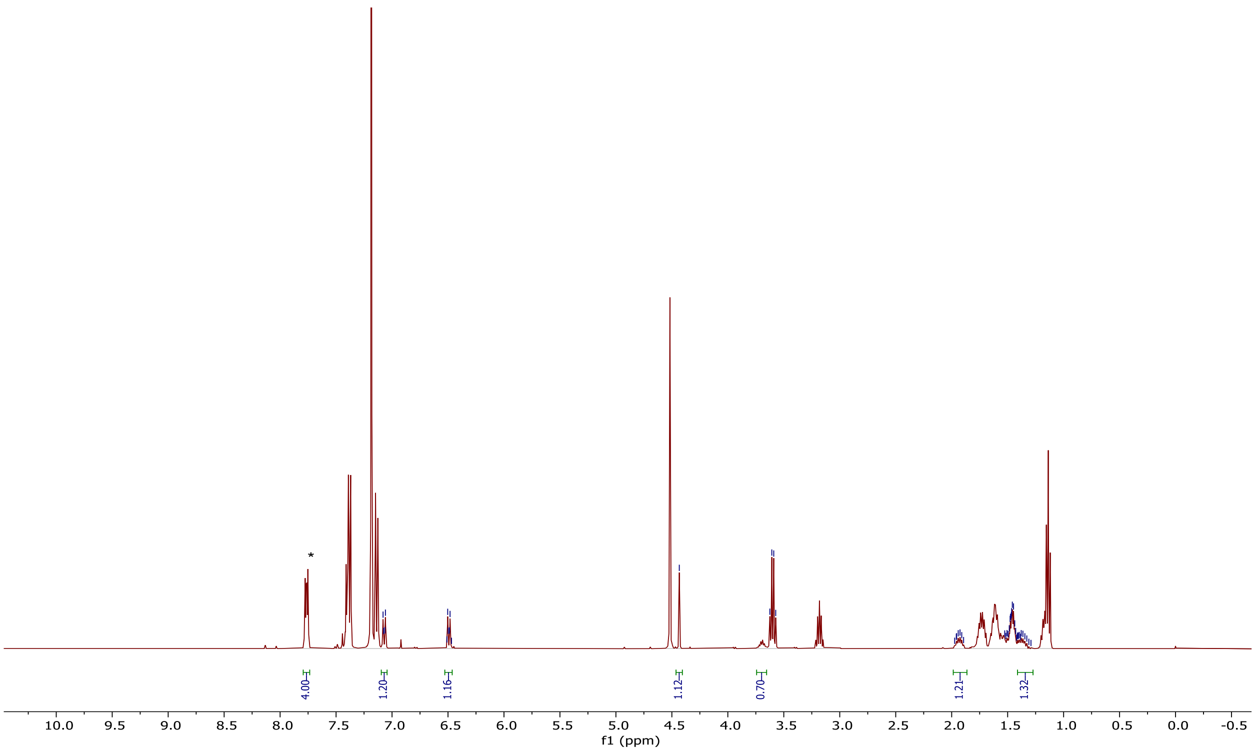

Figure S35 ^{1}H NMR of **3q** (crude product with naphthalene standard peaks labelled with *).

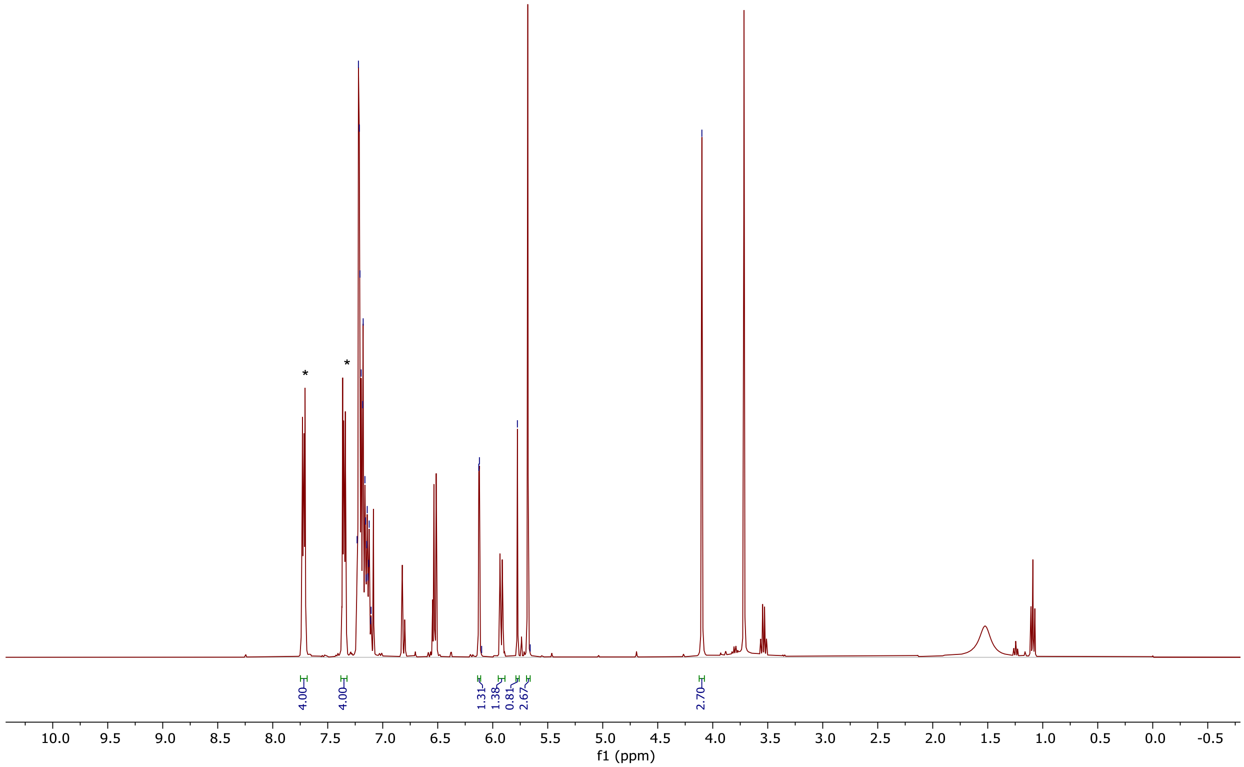

Figure S36 ^{1}H NMR of **3r** (crude product with naphthalene standard peaks labelled with *).

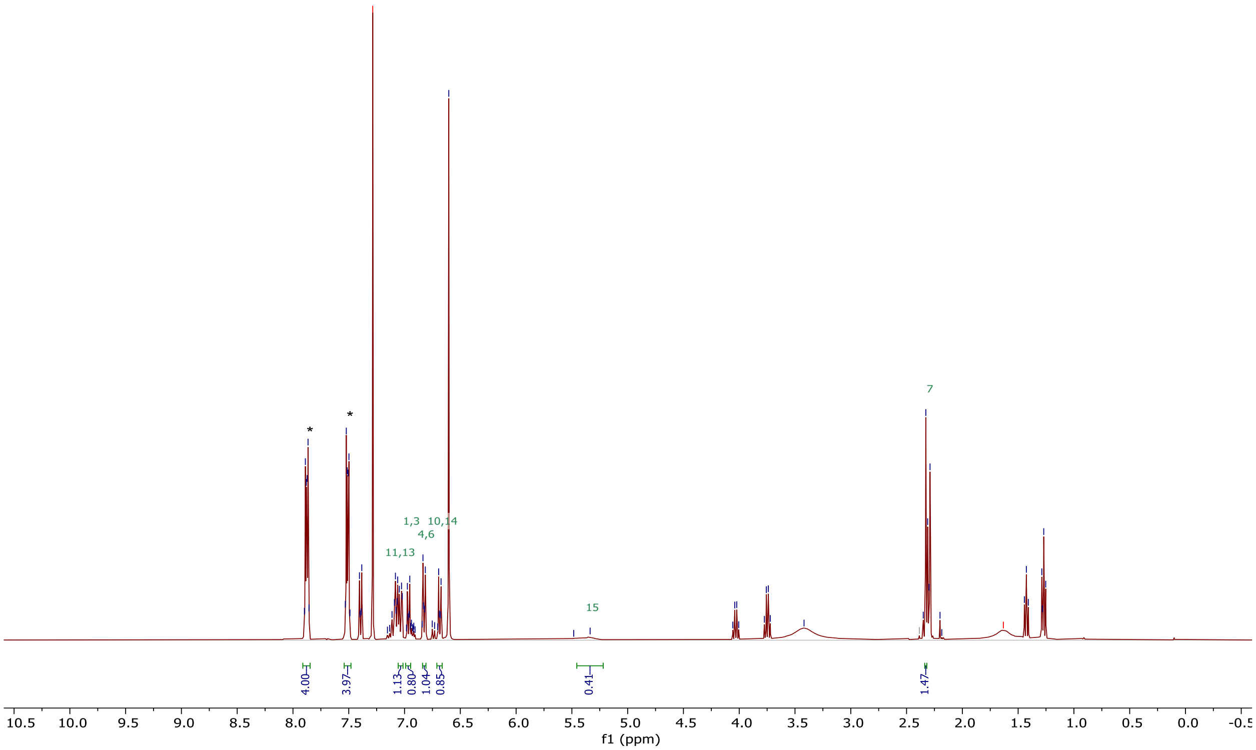

Figure S37 ^{1}H NMR of **3s** (crude product with naphthalene standard peaks labelled with *).

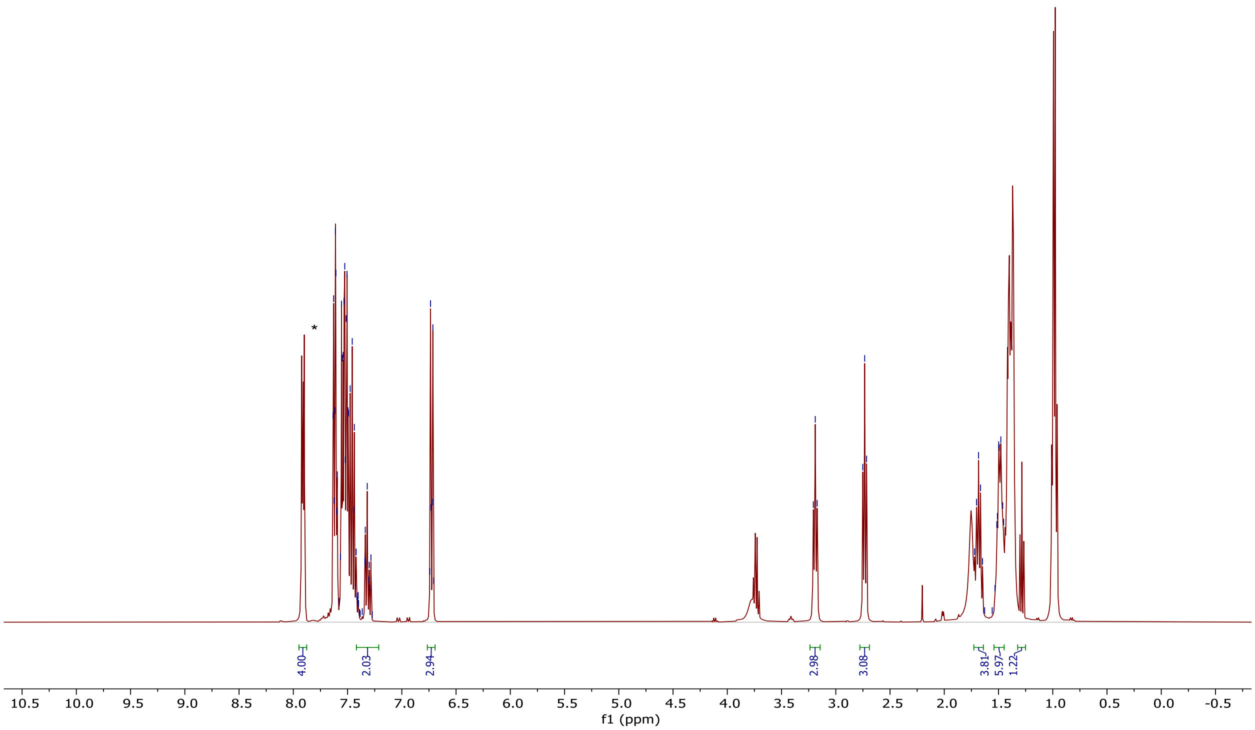

Figure S38 ^{1}H NMR of **3t** (crude product with naphthalene standard peaks labelled with *).

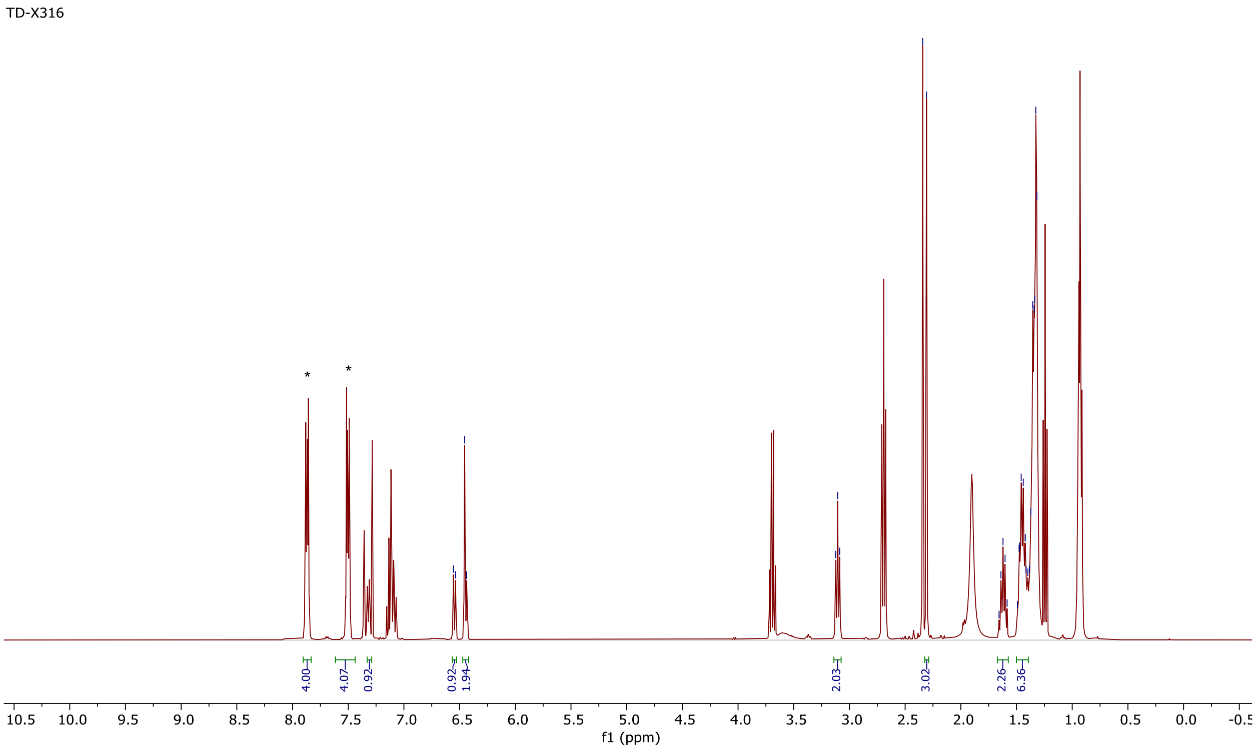

Figure S39 ^{1}H NMR of **3u** (crude product with naphthalene standard peaks labelled with *)

4 References

1. J. Gao, S. Bhunia, K. Wang, L. Gan, S. Xia and D. Ma, *Org Lett*, 2017, **19**, 2809–2812.
2. S. Bhunia, S. V. Kumar and D. Ma, *The Journal of Organic Chemistry*, 2017, **82**, 12603–12612.
3. J. U. Maheswari, C. Krishnan, S. Kalyanaraman and P. Selvarajan, *Physica B: Condensed Matter*, 2016, **502**, 32–38.
4. W. Zhou, M. Fan, J. Yin, Y. Jiang and D. Ma, *Journal of the American Chemical Society*, 2015, **137**, 11942–11945.
5. X. Ma and R. P. Davies, *Advanced Synthesis & Catalysis*, 2022, **364**, 2023–2031.
6. M. Stradiotto and P. MacQueen, *Synlett*, 2017, **28**, 1652–1656.
7. J. W. Park and Y. K. Chung, *ACS Catalysis*, 2015, **5**, 4846–4850.
8. R. Ranjan, A. Chakraborty, R. Kyarikwal, R. Ganguly and S. Mukhopadhyay, *Dalton Trans*, 2022, **51**, 13288–13300.
9. K. Matsumoto, Y. Toubaru, S. Tachikawa, A. Miki, K. Sakai, S. Koroki, T. Hirokane, M. Shindo and M. Yoshida, *The Journal of Organic Chemistry*, 2020, **85**, 15154–15166.
